# Organization of the *Escherichia coli* Chromosome by a MukBEF Axial Core

**DOI:** 10.1016/j.molcel.2020.02.003

**Published:** 2020-04-16

**Authors:** Jarno Mäkelä, David J. Sherratt

**Affiliations:** 1 Department of Biochemistry, University of Oxford, Oxford OX1 3QU, UK

**Keywords:** SMC, MukBEF, loop extrusion, chromosome organization, MatP, *Escherichia coli*

## Abstract

Structural maintenance of chromosomes (SMC) complexes organize chromosomes ubiquitously, thereby contributing to their faithful segregation. We demonstrate that under conditions of increased chromosome occupancy of the *Escherichia coli* SMC complex, MukBEF, the chromosome is organized as a series of loops around a thin (<130 nm) MukBEF axial core, whose length is ∼1,100 times shorter than the chromosomal DNA. The linear order of chromosomal loci is maintained in the axial cores, whose formation requires MukBEF ATP hydrolysis. Axial core structure in non-replicating chromosomes is predominantly linear (1 μm) but becomes circular (1.5 μm) in the absence of MatP because of its failure to displace MukBEF from the 800 kbp replication termination region (*ter*). Displacement of MukBEF from *ter* by MatP in wild-type cells directs MukBEF colocalization with the replication origin. We conclude that MukBEF individualizes and compacts the chromosome lengthwise, demonstrating a chromosome organization mechanism similar to condensin in mitotic chromosome formation.

## Highlights

• MukBEF forms a chromosome axial core dependent on ATP hydrolysis
• MukBEF compacts the chromosome lengthwise while avoiding links between replichores
• MatP determines the shape of the axial core by displacing MukBEF from *ter*
• The displacement by MatP directs MukBEF colocalization with the replication origin

## Graphical Abstract

**Figure:**
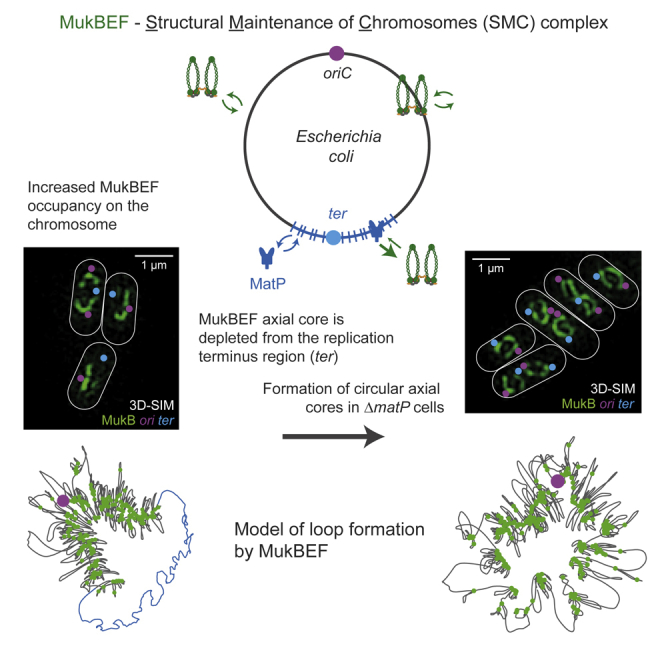

## Teaser

Mäkelä and Sherratt demonstrated that the MukBEF SMC complex organizes the *Escherichia coli* chromosome by forming an axial core, depending on ATP hydrolysis, from which loops of size 20–50 kb emanate. MukBEF enrichment with the replication origin is directed by MukBEF displacement from the replication termination region, directed by MatP.

## Introduction

In all domains of life, structural maintenance of chromosomes (SMC) complexes act on chromosomes, thereby contributing to their faithful propagation and inheritance over generations. SMC roles include individualization, compaction, segregation, and cohesion of sister chromosomes (Hassler et al., 2018, Uhlmann, 2016). A substantial body of work indicates that SMC complexes from diverse organisms topologically entrap DNA double helices into sub-compartments of the complex (Chapard et al., 2019, Hassler et al., 2018, Uhlmann, 2016). Transitions between SMC complex conformational states, powered by ATP hydrolysis, potentially allow the formation of DNA loops by capture of two segments of DNA or, alternatively, by progressive enlargement of a DNA loop from an initial stem, referred as loop extrusion (Alipour and Marko, 2012, Diebold-Durand et al., 2017, Nasmyth, 2001). Although DNA loop formation appears to underlie the action of these molecular machines, exactly how they contribute to chromosome organization remains uncertain (Hassler et al., 2018, Uhlmann, 2016).

**Figure 1:**
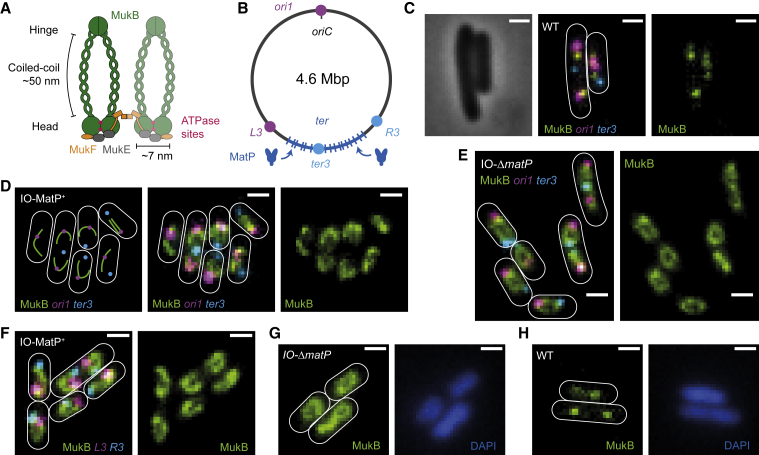
MukBEF Architecture and Function (A) MukBEF architecture showing a functional dimer of dimers complex. (B) *E. coli* chromosome showing 800 kbp *ter* region with *matS* sites (blue bars) that are bound by MatP. Locations of *ori1*, *ter3*, *L3*, and *R3* markers are shown. (C–H) Representative fluorescence images with cell borders of (C) WT cells with labeled MukB, *ori1*, and *ter3*; (D) increased MukBEF occupancy (IO) cells with *ori1* and *ter3* (schematic on left); (E) IO-Δ*matP* cells with *ori1* and *ter3*; (F) IO cells with *L3* and *R3*; (G) IO-Δ*matP* cells with DAPI (4′,6-diamidino-2-phenylindole)-stained nucleoids; and (H) WT cells with DAPI-stained nucleoids. Scale bars: 1 μm.

MukBEF, the *Escherichia coli* (*E. coli*) SMC complex homolog, exhibits the distinctive SMC complex architecture, where MukB forms dimers, each of the monomers consisting of an ABC-type ATPase head domain and a dimerization hinge separated by a long (∼50 nm) antiparallel coiled-coil region (Nolivos and Sherratt, 2014). In contrast to eukaryotic SMCs, which form heterodimers, MukB and other bacterial SMCs are homodimers. The MukB globular ATPase heads are joined by C- and N-terminal interactions of MukF kleisin with the base of the MukB head (“cap”) and the MukB “neck,” a region of coiled-coil adjacent to the head, respectively (Figure 1A) (Zawadzka et al., 2018). Engagement of the heads and MukF are required for MukB ATPase activity (Zawadzka et al., 2018) and MukBEF function *in vivo* (Badrinarayanan et al., 2012a). A distinguishing feature of MukF kleisins is that they form dimers through an N-terminal winged-helix domain, leading to the formation of dimer of dimer MukBEF complexes *in vivo* and *in vitro* (Badrinarayanan et al., 2012a, Fennell-Fezzie et al., 2005, Nolivos and Sherratt, 2014, Rajasekar et al., 2019, Zawadzka et al., 2018). Impairment of MukF dimerization leads to a failure of MukBEF complexes to stably associate with the chromosome, defects in chromosome segregation, and anucleate cell production, similar to the phenotype of cells lacking MukBEF subunits (Danilova et al., 2007, Niki et al., 1991, Rajasekar et al., 2019). Finally, MukE is an essential accessory (KITE) protein that binds MukF and modulates MukB ATPase activity (Palecek and Gruber, 2015, Zawadzka et al., 2018).

MukBEF homologs, containing a dimeric kleisin, are confined to γ-proteobacteria and have co-evolved with a set of genes, including MatP, which binds to ∼23 *matS* sites in the ∼800 kb chromosome replication terminus region (*ter*) (Figure 1B) (Brézellec et al., 2006, Mercier et al., 2008). Interaction of MukBEF with MatP-*matS* leads to displacement of MukBEF complexes from *ter* (Nolivos et al., 2016). Furthermore, MukB interacts with the chromosome decatenase, topoisomerase IV (TopoIV), providing a functional link between chromosome organization and unlinking (Hayama and Marians, 2010, Li et al., 2010, Nolivos et al., 2016).

Chromosome-bound MukBEF complexes form clusters, observed as “foci” by fluorescence microscopy (Figure 1C) (Badrinarayanan et al., 2012a, Danilova et al., 2007, Nolivos et al., 2016). These clusters position replication origin (*oriC*) regions to either mid-nucleoid (newborn cells) or nucleoid quarter positions (cells that have replicated and segregated their *oriC* regions), with the MukBEF clusters being positioned on the nucleoid by a Turing patterning mechanism (Badrinarayanan et al., 2012a, Badrinarayanan et al., 2012b, Hofmann et al., 2019, Murray and Sourjik, 2017). ∼50% of ∼100 MukBEF complexes in a cell are tightly associated with DNA, while ∼20% of these are present in foci (Badrinarayanan et al., 2012a).

We demonstrate that after modest overexpression from the endogenous chromosomal locus, MukBEF complexes form a thin axial core to the chromosome. The formation of MukBEF axial cores is directed by ATP-hydrolysis-dependent reactions, since a MukB mutant that binds ATP but is impaired in hydrolysis formed *ter*-associated “foci” rather than axial cores, while overexpression of a MukB mutant that cannot bind ATP led to dispersed fluorescence throughout the cell because it fails to associate stably with chromosomes. Analysis of genetic loci with respect to the axial core indicates that the overall linear order is retained, and the chromosome is organized uniformly about the axial core, leading to the conclusion that DNA loops must emanate from the axial core. The shape of the axial core is determined by MatP as it displaces MukBEF from *ter*, transforming a prospective circular axial core into a linear one. Abrogation of MukBEF displacement by *matP* deletion led to formation of the circular axial core. The axial core is ∼1,100-fold shorter than the length of the chromosomal DNA with no detectable differences to wild-type (WT) in overall chromosome morphology. Under these conditions, cells grew normally and produced no anucleate cells, indicating no impairment of chromosome segregation. To reveal a possible mechanism that would give rise to the observed structures, we used stochastic modeling and simulated the action of MukBEF as a loop-extruding machine. Our simulations generated linear or circular structures without MukBEF displacement from *ter* on the chromosome that recapitulated the ones from microscopy. Finally, we showed that the MukBEF displacement from *ter* can generate a colocalization gradient of chromosomal MukBEF complexes, whose distance from *oriC* is minimized and maximized from *ter*, as observed by microscopy in WT cells. We propose that MukBEF displacement from *ter* acts as an alternative mechanism to promote SMC-*oriC* association.

## Results

### MukBEF Forms an Axial Core to the Chromosome

To address how MukBEF organizes the *E. coli* chromosome, and why MukBEF clusters colocalize with and position the *oriC* region (Figures 1A–1C) (Badrinarayanan et al., 2012a, Badrinarayanan et al., 2012b, Danilova et al., 2007, Hofmann et al., 2019, Nolivos et al., 2016), we modestly overexpressed MukBEF from the endogenous *mukBEF* operon by replacing the native promoter with an inducible promoter, *P*_*ara*_. We used a strain with a functional mYpet fusion to the endogenous *mukB* gene and FROS (Fluorescent Repressor-Operator-System) markers located near *oriC* (*ori1*) and close to the center of *ter* (*ter3*) (Figure 1B) (Nolivos et al., 2016). Cells grown under constant presence of 0.2% arabinose resulted in 6.3 ± 0.4-fold overexpression (±SEM) (Figure S1). Under the conditions of increased MukBEF occupancy (IO) on the chromosome, fluorescent MukBEF formed a filamentous axial core that was predominantly linear (Figures 1D and S2). The *ter3* marker was depleted of MukBEF fluorescence, demonstrating that the linearity of the axial core is a direct consequence of the MatP-*matS*-dependent displacement of MukBEF from *ter*. Confirming this interpretation, Δ*matP* cells had predominantly circular axial cores (Figure 1E). Unlike SMCs in many bacterial species (for example, Le et al., 2013, Wang et al., 2017), MukBEF does not link chromosome arms together; instead, the ends of linear axial cores in cells with MatP present (MatP^+^) localized near *L3* and *R3* markers that flank the *ter* region (Figures 1B and 1F). As *ori1* is localized halfway through the axial core (Figure 1D) and *ter3* is in the MukBEF-depleted region, this indicates that the MukBEF axial cores retain the linear order of the chromosome.

The MukBEF axial cores form a proteinaceous structure on the chromosome and require DNA to act as a scaffold, since a mutant deficient in DNA binding as a consequence of a failure to bind ATP, MukB^D1406A^EF (Badrinarayanan et al., 2012a), did not form a structure after overexpression (Figure S1). The formation of axial cores is also dependent on ATP hydrolysis, since a MukB^E1407Q^EF mutant that binds ATP and loads on the chromosome but is impaired in hydrolysis (Nolivos et al., 2016) formed chromosome-associated foci near *ter3* under increased occupancy, rather than axial cores (Figure S1). MukBEF IO is not detrimental to the cells, as we observed the same generation time in IO cells as in WT cells (Figure S1) and IO cells did not produce anucleate cells, which arise as a consequence of a failure in chromosome segregation, a hallmark of MukBEF defects (Figure S1). DAPI-stained nucleoids of IO cells showed no detectable morphological differences to those of WT cells (Figures 1G, 1H, and S3), consistent with overall chromosome compaction being unaffected by IO. MukBEF axial cores were also observed in transcription-inhibited cells (Nonejuie et al., 2013) and in cells of increased volume after treatment with A22 (Figure S4) (Wu et al., 2019), demonstrating that formation of the axial core occurs with less molecular crowding or a different cellular shape and volume.

**Figure 2:**
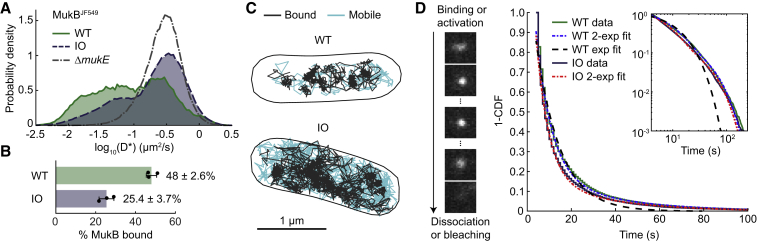
Single MukBEF Molecules on the Chromosome (A) Log-scale distribution of apparent diffusion coefficients (D^∗^) from single-molecule tracking of MukB^JF549^ with 15 ms exposure time in WT (38,502 tracks), IO (355,560 tracks) and Δ*mukE* (166,991 tracks) cells. Data from 3 repeats. (B) Percentage of molecules classified as bound (D^∗^ < 0.0875 μm^2^/s) in WT and IO cells. The threshold was defined by the lower 5% quantile of D^∗^ in Δ*mukE*. Data from (A). Error bars denote SD. (C) Representative map of MukB^JF549^ tracks in WT (top) and IO (bottom) cells. (D) Residency time of MukB^JF549^ molecules on the chromosome. Example frames with 1 s exposure time of a molecule producing a clear spot when bound until it dissociates or bleaches. Survival probability distributions (1-CDF) of measured residency times in WT (10,084 tracks) and IO (14,734 tracks). The data were fitted by a double-exponential function (for comparison, an exponential fit to WT). Inset shows log-log plot of the same data. The blinking- and bleaching-corrected residency time for WT is 66.9 ± 15.3 s and for IO is 63.7 ± 13.5 s (±SEM from 4 experiments).

To estimate the number of bound MukBEF complexes forming the axial cores, we first measured the fraction of chromosome-bound MukBEF complexes using single-molecule tracking with a functional HaloTag fusion to the endogenous *mukB* gene (Figures 2A–2C) (Banaz et al., 2019). IO cells had 25.4 ± 3.7% (±SD) of molecules immobile (chromosome-associated), as compared to 48 ± 2.6% (±SD) molecules in WT cells. Consequently, the occupancy of bound MukBEF complexes on chromosomes, given the 6.3-fold overexpression, was increased 3.3-fold as compared to WT cells. Together with previously measured MukBEF numbers in cells (Badrinarayanan et al., 2012a), we estimate ∼53 and ∼175 dimer of dimers MukBEF complexes on the chromosome in WT and IO cells, respectively. Additionally, using single-molecule tracking of MukB^JF549^ with a 1 s exposure time, we determined almost identical residency times for the chromosome-associated MukBEF complexes in WT (64 ± 14 s) and IO cells (67 ± 15 s) (±SEM) (Figure 2D), similar to the estimate for WT cells using FRAP (Fluorescence Recovery After Photobleaching) (Badrinarayanan et al., 2012a).

### Association of MukBEF Axial Cores with Chromosomal Loci

**Figure 3:**
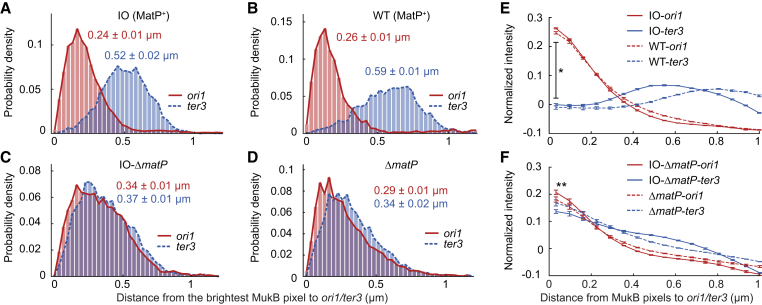
Quantitative Analyses of MukBEF Localization (A–D) Distances between the brightest MukB pixel and *ori1*/*ter3* markers (±SEM) in (A) increased MukBEF occupancy (IO) (5,463 cells), (B) WT (4,240 cells), (C) IO-Δ*matP* (4,483 cells), and (D) Δ*matP* (4,702 cells) cells. Prior to imaging, cells were treated with serine hydroxamate; only cells with a single chromosome were included in the analysis. Data from 3 repeats. (E and F) Normalized MukB pixel intensity as a function of distance to *ori1*/*ter3* in asynchronous populations of (E) MatP^+^ and (F) Δ*matP* cells with WT and increased MukBEF occupancy (WT, 11,567; IO, 11,030; Δ*matP*, 13,089; IO-Δ*matP*, 8,392 cells). ^∗^ and ^∗∗^ denote two-sample t test between background corrected MukB intensity at *ori1* and *ter3* for WT (p value 0.0042), Δ*matP* (p value 0.5164), IO (p value 0.0039), and IO-Δ*matP* (p value 0.0996) cells. Error bars denote SEM from 3 repeats.

To quantitatively analyze MukBEF axial cores in relation to genetic markers, we enriched for cells with completely replicated chromosomes by incubation with serine hydroxamate, thereby avoiding bias from partially replicated chromosomes. During the treatment, cells do not initiate new rounds of replication, but most complete any ongoing rounds (Ferullo et al., 2009). In single chromosome WT and IO MatP^+^ cells, the brightest MukBEF locus showed stronger colocalization with *ori1* than *ter3* as shown by the distances (Figures 3A and 3B), whereas there was no difference in colocalization of MukBEF with *ori1* and *ter3* in Δ*matP* cells (Figures 3C and 3D). Therefore, even though the MukBEF foci in WT cells are replaced by the axial cores after IO, association of MukBEF with *ori1* remains. In contrast, circular axial cores in IO-Δ*matP* cells were uniform in relation to *ori1*/*ter3* loci (Figure 3C). A uniform occupancy excludes the possibility of specific MukBEF loading sites near *oriC* or *ter*, since the short MukBEF residency times on the chromosome would result in an intensity gradient. Moreover, *ori1* and *ter3* were observed in opposite positions along the circular axial core, indicating that the replichores are organized into the axial core uniformly (Figures 1E and S5). Finally, we measured radial MukBEF intensity in circular axial cores in IO-Δ*matP* cells to show that the overall MukBEF occupancy in the axial core is uniform, though individual axial cores are inherently more variable (Figure S5). Together, these findings demonstrate that as MukBEF forms axial cores, it loads onto and organizes the chromosome uniformly, with the exception of MatP-*matS* occupied *ter*.

To assess MukBEF chromosome association in replicating cells, where *ori1* number exceeds *ter3* number due to partially replicated chromosomes, we quantified all chromosome-associated MukBEF complexes in relation to *ori1* and *ter3*. To compare the profiles with different expression levels of WT and IO cells, we subtracted the average pixel intensity and normalized by the maximum intensity and subsequently measured distances to the closest *ori1* and *ter3* (Figures 3E and 3F). In MatP^+^ cells, the MukBEF intensity was highest in the vicinity of *ori1*, ∼0.5 μm away from *ter3*, while Δ*matP* cells exhibited similar preference for both *ori1* and *ter3* with a descending intensity profile from the chromosome toward the cell periphery. Importantly, the profile patterns were almost identical in IO cells and in normal-occupancy cells. We also measured the intensity profiles during induction of the MukBEF overexpression, as cells transition from distinct foci into a continuous axial core (Figure S3). The intensity curves were unchanged during induction and were similar to cells with fully induced MukBEF expression. These observations indicate that the nature of MukBEF association with chromosomes is unaffected by increased MukBEF occupancy; while less of the chromosome is occupied by MukBEF complexes in WT cells, the probability of MukBEF occupying a chromosome locus relative to other loci remains the same.

### MukBEF Axial Core Dimensions

**Figure 4:**
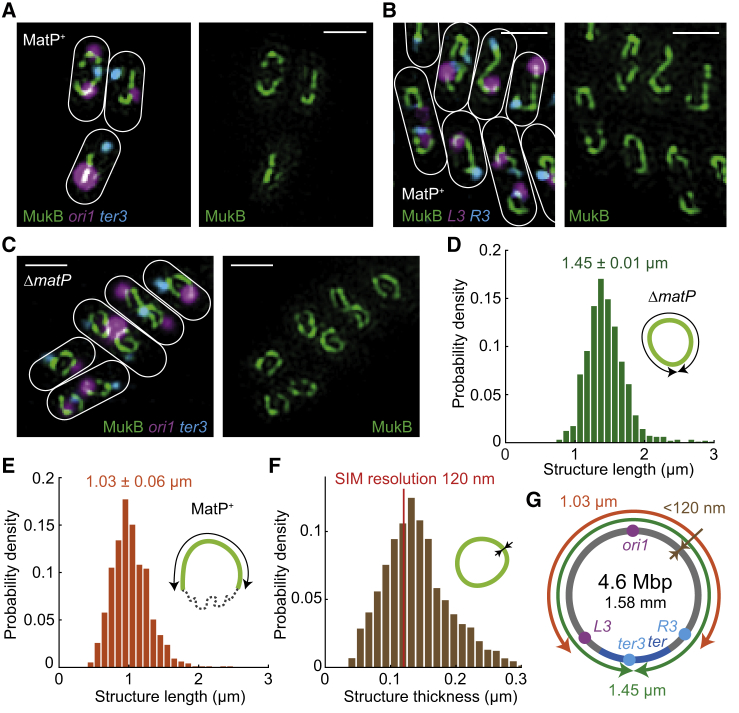
3D-SIM Analysis of MukBEF Axial Cores (A–C) Representative SIM images of increased MukBEF occupancy (IO) in (A) MatP^+^ with *ori1* and *ter3*, (B) MatP^+^ with *L3* and *R3*, and (C) Δ*matP* with *ori1* and *ter3* cells. 3D-SIM images were projected onto 2D for visualization. Scale bars: 1 μm. (D) Distribution of axial core contour lengths (n = 986, ± SEM) measured from IO-Δ*matP* cells. (E) Distribution of linear axial core lengths (n = 971, ± SEM) measured from IO cells. (F) Distribution of MukBEF axial core thicknesses (n = 6,657). The peak is at 132 nm. Red line denotes the resolution of SIM. For all panels, prior to imaging, cells were treated with serine hydroxamate to prevent replication initiation. Data from 3 repeats. (G) Schematic of MukBEF axial core dimensions.

To gain more insights into the nature of the axial core, we used three-dimensional structured illumination microscopy (3D-SIM) in live cells (Figures 4A–4C and S4; Videos S1 and S2). Given the 2-fold improvement in both lateral and axial resolution (Kraus et al., 2017), 3D-SIM imaging facilitated quantification of the dimensions of the axial cores and estimation of the lengthwise compaction, i.e., reduction of effective contour length of the chromosome by formation of chromosomal DNA loops by MukBEF. As before, we enriched for cells with completely replicated chromosomes by incubation with serine hydroxamate, and the dimensions of the axial cores were quantified in cells with completely replicated chromosomes. We measured the contour length along the centerline of the circular axial core in IO-Δ*matP* cells to be 1.45 ± 0.01 μm (±SEM) (Figure 4D). Given that the 4.64 Mbp circular chromosome of *E. coli* has a contour length of 1.58 mm, this corresponds to a ∼1,100-fold lengthwise compaction along the chromosomal axis. Additionally, we measured the length of the linear axial core along the centerline in IO cells to be 1.03 ± 0.06 μm (±SEM) (Figure 4E), shorter than in IO-Δ*matP* cells (two-sample t test, p value 0.0028), extending from genetic markers *L3* to *R3* (3.22 Mbp; 69.5% of the genome). Since *L3* and *R3* colocalize with the ends of the axial core, the displacement of MukBEF extends beyond the outer *matS* sites and *ter*. The lengthwise compaction in the linear axial cores (∼1,100-fold) was found similar to that of the circular cores in IO-Δ*matP* cells. Finally, we determined the thickness of the axial cores. The thickness (FWHM) was approximately ∼130 nm (Figure 4F), close to the limit of SIM resolution (∼120 nm), thereby suggesting that the actual thickness is likely less and of the same order as the dimensions of functional MukBEF complexes (Figure 1A). In combination with the estimated number of MukBEF dimer of dimers on the chromosome with the increased occupancy (∼175), we infer that there is a complex for every ∼6 nm of axial core length, consistent with it being a near continuous array of MukBEF complexes. Moreover, the average length of DNA associated with each MukBEF dimer of dimer is ∼22 kbp. We summarize the dimensions of MukBEF axial cores in Figure 4G.

Video S1. 3D Rendering of MukB-mYpet 3D-SIM Data from MukBEF Increased Occupancy Δ*matP* Cells, Related to Figure 4 Scale bar: 1 μm.

Video S2. 3D Rendering of MukB-mYpet 3D-SIM Data from MukBEF Increased Occupancy MatP^+^ Cells, Related to Figure 4 Scale bar: 1 μm.

In MatP^+^ cells, MukBEF complexes are displaced from the region that extends from *L3* and *R3* markers, including the 800 kbp *ter* region. To estimate the relative compaction of the MukBEF-displaced region, we measured the minimal distance between *L3* and *R3* markers (1.42 Mbp; Figure 1B) in asynchronous IO MatP^+^ cells. We observed a bimodal distribution of distances (Figure S3), indicating that this region can be either more loosely (∼400-fold), or densely (∼1,000-fold) compacted. This is consistent with the observation that different genetic markers in *ter* can localize to distant regions of the same cell (Wang et al., 2005), with Hi-C data (Lioy et al., 2018), and with analysis of cells with increased volume (Wu et al., 2019). The *L3*-*R3* marker distance in WT and IO MatP^+^ cells was indistinguishable, but in Δ*matP* cells, where MukBEF complexes are not displaced from *ter*, *L3* and *R3* markers are less separated than in WT (Figure S3), showing that MukBEF action reduces flexibility in *ter*. As such, MatP has an important role in directing the chromosome arms to different cell halves in *E. coli*.

### Stochastic Models of MukBEF Loop Formation on the Circular Chromosome

**Figure 5:**
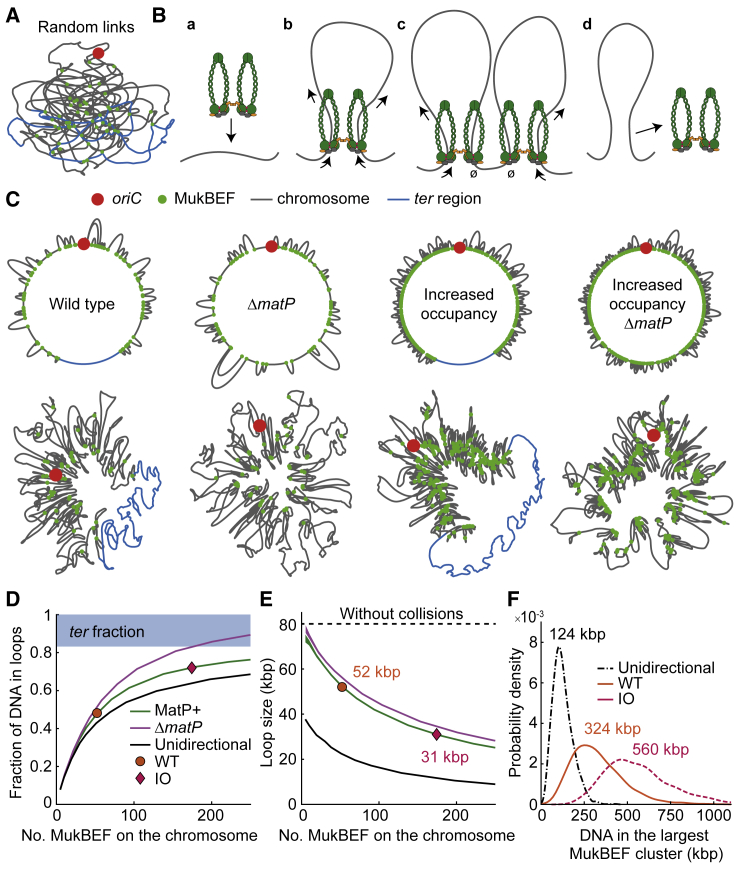
Modeling Loop Extrusion by MukBEF (A) Example of a simulated randomly linked *E. coli* chromosome. (B) Description of the model: (a) A MukBEF dimer of dimers associates at a random site on the chromosome. (b) The loop is enlarged by the two dimers moving in opposite directions along the chromosome. (c) Collisions between MukBEFs prevent loop extrusion only on the internal collided dimers. (d) MukBEF spontaneously dissociates, releasing the loop. The MatP-bound *ter* region immediately displaces MukBEF upon contact. (C) Representative *E. coli* chromosomes with and without MukBEF displacement from *ter* and WT or increased MukBEF occupancy. (top) Beginning and end of loops with MukBEF (green dots) along the chromosome. (bottom) Force-directed layouts of the chromosomes. (D and E) (D) Fraction of the chromosome within a loop and (E) loop size per individual MukBEF as a function of the number of chromosome-bound MukBEF dimers of dimers. In unidirectional loop extrusion, dimers are not connected and each dimer acts independently, binding and extruding a loop in a randomly chosen direction. Estimated numbers for MukBEF for WT and IO cells are shown. Line thickness denotes 95% bootstrap confidence interval for the mean across 1,000 simulation replicas. (F) Distribution of DNA in the largest MukBEF cluster (no unlooped DNA between them) for WT and IO occupancy and unidirectional loop extrusion (from the same data as in E).

How does MukBEF form the chromosome axial core? Although DNA loop formation appears to underlie the action of SMC complexes and homologs, exactly how the loops are formed remains controversial (Hassler et al., 2018, Uhlmann, 2016). Random capture of two DNA segments by MukBEF cannot generate the observed axial cores (Figure 5A). As a model, progressive enlargement of DNA loops, i.e., loop extrusion, promotes linear chromosome organization, individualization of the chromosome arms, and formation of a brush-like chromosome structure (Goloborodko et al., 2016). To understand how loop extrusion could lead to the formation of the MukBEF axial cores, we undertook stochastic simulations of the process using experimentally derived MukBEF parameters. We modeled MukBEF complexes as dimers of dimers that load randomly onto the chromosome and are capable of extruding loops bidirectionally at a rate of 600 bp/dimer/s (for comparison, rates of 100 bp/s and 1,500 bp/s were also used) (Figures 5B and S6) (Ganji et al., 2018). We assume that dimers of MukBEF complexes cannot overtake each other, and when loop extrusion brings two translocating complexes together, the inner dimers stall while the outer dimers continue to extrude loops. To end the loop extrusion, MukBEF complexes spontaneously dissociate from chromosomes after an exponential dwell time measured here (65 s), while any complexes that encounter MatP-*matS* in *ter* are immediately released from DNA. The loading rate was adjusted to reproduce the measured (48%) percentage of chromosome-bound MukBEF complexes in WT.

We show representative simulations with (MatP^+^) and without (Δ*matP*) MukBEF displacement from *ter* and with WT or IO numbers of bound molecules on the chromosome (Figure 5C). The chromosomes are visualized as a circular chromosome map with MukBEF-generated loops and as a force-directed layout of the chromosome that mimics “folding” of the chromosome given MukBEF-formed loops. WT MukBEF numbers results into an average loop size of 52 kbp, and the loops contain half of the chromosome (WT 48%). As MukBEF occupancy on the chromosome increases, more of the chromosome is included into MukBEF-generated loops (IO 72%) (Figure 5D), while the loop size in individual MukBEF complexes decreases (IO 30 kbp) due to more frequent collisions (Figure 5E). The colliding MukBEF complexes form continuous clusters that can contain up to 750 kbp of DNA with WT occupancy or >1 Mbp with IO (Figures 5F and S6). By comparison, the simulations found unidirectional loop extrusion inefficient, especially in formation of larger clusters, due to gaps left behind (Figures 5D, 5F, and S6), as inferred elsewhere (Banigan and Mirny, 2019). The requirement for MukBEF to act as dimers of dimers provides a plausible mechanism for bidirectional loop extrusion (Badrinarayanan et al., 2012a, Rajasekar et al., 2019). Experimentally, the MukBEF axial core is significantly more lengthwise compacted (∼1,100-fold) than the simulations of MukBEF loop extrusion predict (∼10-fold). This is a consequence of the measured relatively short residency times of MukBEF complexes (∼65 s); greater lengthwise compaction in the simulations would require much longer residency times. as a higher loop extrusion rate of 1,500 bp/s does not significantly affect the lengthwise compaction (Figure S6). Therefore, we propose that while MukBEF is responsible for forming the chromosome axial core that can act as a scaffold for DNA loop formation, other proteins capable of forming DNA loops or indirectly influencing chromosome compaction contribute considerably to the overall lengthwise compaction (Dillon and Dorman, 2010, Lioy et al., 2018).

### MukBEF Displacement from *ter* Promotes Its Colocalization with *oriC*

**Figure 6:**
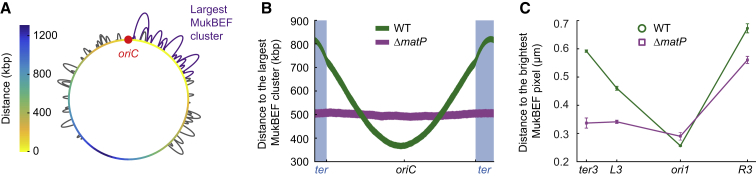
MukBEF Colocalization with Chromosomal Loci (A) Illustration of the shortest distance from a chromosome locus to the largest MukBEF cluster (no unlooped DNA between complexes) along the chromosome. Color map denotes the distance in kbp from loci along the circular chromosome. The largest MukBEF cluster is marked with violet loops. (B) Shortest distance from a chromosome locus to the largest MukBEF cluster with WT occupancy and with (WT) or without (Δ*matP*) MukBEF displacement from *ter*. Line thickness denotes 95% bootstrap confidence interval for the mean across 2,000 simulation replicas. (C) Measured distance between the brightest MukB pixel and *ori1*, *ter3* (WT, 4,240 cells; Δ*matP*, 4,702 cells), *L3*, and *R3* (WT, 2,376 cells; Δ*matP*, 2,706 cells) markers. Prior to imaging, cells were treated with serine hydroxamate, and only cells with a single chromosome were included in the analysis. Error bars denote SEM from 3 repeats.

It has been proposed that MukBEF clusters are positioned autonomously at fixed positions on the nucleoid by a Turing patterning mechanism, and non-trivial interactions between MukBEF and *oriC* regions subsequently position *oriC* (Badrinarayanan et al., 2012b, Hofmann et al., 2019, Murray and Sourjik, 2017). The requirement for the accurate positioning of *oriC*, and consequently other chromosomal loci, is colocalization with MukBEF clusters. However, the mechanism for the observed colocalization remains unknown, given that no specific MukBEF binding sites in *oriC* or in the vicinity have been found. With the MukBEF loop extrusion model presented here, we considered whether it explains the colocalization in WT cells (in IO cells, *ori1* is always localized halfway through the axial core) (Figure 3B) (Danilova et al., 2007). We used the largest MukBEF cluster as a proxy for the brightest MukBEF foci and computed the shortest distance along the model chromosome between a locus and the largest MukBEF cluster with WT occupancy (Figure 6A). DNA loops formed by MukBEF shorten the distances by bridging distant chromosome segments. The results show a minimum distance between the largest MukBEF cluster and the *oriC* region, from which the distance gradually increases until reaching the maximum distance in the middle of the *ter* region (Figure 6B). This is a direct consequence of MatP-*matS* depleting MukBEF from *ter* (Figure S6), which is diametrically opposite to *oriC*. Concomitantly, *oriC* is in the center of the MukBEF-occupied region, and the minimum distance always forms opposite of the depletion region. The absence of MukBEF displacement from *ter* (Δ*matP*) abrogates this effect, resulting in uniform distances with all chromosome loci (Figure 6B). The profiles resemble the radial intensity patterns in IO-MatP^+^ or Δ*matP* cells showing the overall MukBEF occupancy along the chromosome (Figure S5). Different loop extrusion rates (100 bp/s and 1,500 bp/s/dimer) recapitulated the overall patterns, albeit with different overall distances (Figure S6).

In agreement with the simulations, WT cell distances from the brightest MukBEF foci were shortest at *ori1* and longer at *ter3* (Figure 6C). *L3* showed an intermediate distance as predicted by the model. Unexpectedly, distances to *R3* were greater than to *ter3*. Although we do not know the reason for this, it is likely to be a consequence of the behavior of FROS marker itself, as a similar effect was observed in a previous study of genetic marker positioning (Wang et al., 2006). Nevertheless, as predicted by the simulations, in Δ*matP* cells the distances decreased with all other markers except *ori1*, from which distances actually increased, as predicted by the model. MukBEF clusters colocalized almost equally with *ori1*, *L3*, and *ter3*, concluding that, indeed, MatP-*matS*-directed MukBEF displacement from *ter* can generate the observed *oriC* colocalization pattern (Danilova et al., 2007). We note that the proposed mechanism for the MukBEF-*oriC* colocalization pattern on the chromosome is not dependent on loop extrusion per se, and other linear chromosome organization mechanisms could reproduce the colocalization pattern. The crucial component for forming the colocalization gradient is MukBEF displacement from *ter*, diametrically opposite to *oriC* on the circular chromosome.

## Discussion

By using rigorous analysis of quantitative and super-resolution imaging data, we have demonstrated that under modest increased chromosome occupancy, MukBEF forms a proteinaceous axial core to the *E. coli* chromosome, dependent on MukBEF ATP hydrolysis. The axial core is ∼1,100 times shorter than the length of the chromosomal DNA, with thickness of the same order as individual MukBEF complex dimensions. Because the overall linear order of the chromosome was retained with respect to the axial core, the only possible organization has loops of various sizes emanating from this axial core. These findings suggest strongly that MukBEF has a direct architectural role in *E. coli* chromosome organization. Previously, it has been inferred that MukBEF promotes DNA-DNA contacts in the range of several hundreds of kb (Lioy et al., 2018) and that clusters of its complexes, found at specific locations on the nucleoid, position origins of replication (Badrinarayanan et al., 2012b, Hofmann et al., 2019). Entropic and/or cumulative effects of MukBEF action in chromosome segregation and organization have also been discussed (Jun and Wright, 2010). Nevertheless, there has been no premise that, in addition to any singular MukBEF activity, the emergent behavior of a higher-order structure organizes the chromosome, as revealed here. To form an axial core, sufficient MukBEF density on the chromosome is required, and we expect the MukBEF axial cores in WT cells to be more granular, less continuous entities, but with a comparable level of chromosome compaction in the MukBEF clusters.

MukBEF axial cores are at least superficially similar to “vermicelli” chromosomes observed in mammalian cells when cohesin occupancy was increased by impairing WAPL, which stimulates ATP-hydrolysis-dependent cohesin removal from chromosomes (Tedeschi et al., 2013). Similarly, the axial scaffolds formed by condensin II during the early stages of mitotic chromosome formation resemble those here (Gibcus et al., 2018). We propose that SMC complexes universally form a proteinaceous structure on the chromosome from which DNA loops emanate. Nevertheless, the molecular mechanisms that direct the formation of the structures remain elusive. Stochastic entrapment of DNA double helices, interactions between topologically (or non-topologically) loaded complexes, and convergence of the complexes during loop extrusion can, in theory, all generate the observed SMC scaffolds, and these processes do not have to be mutually exclusive (Cheng et al., 2015, Hassler et al., 2018, Uhlmann, 2016). A simple model of random stepping on a thermally fluctuating DNA, which is consistent with the observations of Ganji et al., (2018), has been presented in Lawrimore et al., (2017). In directing and limiting SMC action to the same DNA molecule, loop extrusion has clear attractions, as it promotes linear chromosome compaction and individualization of the chromosome arms (Goloborodko et al., 2016).

Individualization and separation of chromosome arms by MukBEF contrasts to the situation in *Bacillus subtilis* and *Caulobacter crescentus*, where the action of their SMC complexes zips up the two chromosome arms rather than separating them, resulting in the two chromosome arms being colinear along the cell long axis (Le et al., 2013, Wang et al., 2017). We surmise that the putative SMC-directed loops could still form within a chromosome arm, with higher-order interactions bringing the two arms together. Despite the different outcomes in overall chromosome organization between *E. coli* and *B. subtilis*, the action of their SMC complexes at the molecular level in generating DNA loops is likely to be similar and dependent on ATP hydrolysis (Wang et al., 2018). A possible analogy is the differential roles of cohesin in chromosome organization prior to S phase and its role in linking sisters after replication (Uhlmann, 2016).

The circular chromosome has been imaged in shape-modified *E. coli*, where the larger cellular volumes constrain chromosome conformation less (Wu et al., 2019). In enlarged WT cells, DNA density varied along the chromosome with the lowest density in *ter*, while in enlarged *ΔmatP* cells, the density was found to be more uniform, consistent with our observations of MukBEF axial cores in normal-shaped cells. These observations support our proposal of MukBEF being a main organizer of the chromosome and the role of MatP as a regulator of MukBEF action. The more granular, less continuous WT MukBEF axial core that we propose could be reflected in the observed high-density DNA regions, whose location and number were found to be highly dynamic in shape-modified cells. It would therefore be insightful to analyze the influence of MukBEF impairment on chromosome density in increased-volume cells.

MukBEF action on the chromosome is shaped by the chromosome-associated MatP, which plays a key role in generating the distinctive *E. coli* chromosome organization. First, the displacement of MukBEF from *ter* by MatP promotes association with the *oriC* region. This contrasts with the strategy in most characterized bacteria that preferentially load their SMC complexes at *oriC*-proximal ParB-*parS* sites (Gruber and Errington, 2009, Wang et al., 2017). Our results provide no support for the presence of a MukBEF loading site in the *oriC* region (or indeed elsewhere), since no enrichment was observed in axial core intensity toward *ori1* (or *ter3*) upon *matP* deletion. We propose that MukBEF complexes can load equally well on all regions of the chromosome. Second, by deterring formation of long-range DNA-DNA interactions through MukBEF displacement (Lioy et al., 2018), MatP creates a flexible *ter* domain that can be present in distant regions of the same cell (Wang et al., 2005). In WT *E. coli*, the chromosome arms are directed to different cell halves (Wang et al., 2006), but in Δ*matP* cells, lacking a flexible *ter* region, the replichore separation into opposite cell halves is less pronounced, as illustrated by the smaller distances between *L3* and *R3* markers. Separation of the replichores may minimize the opportunities for chromosome entanglement and knotting, for example, by the inappropriate action of TopoIV. Third, the precocious early separation of newly replicated *ter* in Δ*matP* cells has been proposed to result from the increased abundance of MukBEF and TopoIV in *ter* in these cells (Nolivos et al., 2016). Still, the precise mechanism of MukBEF displacement remains to be uncovered. MatP interacts directly with the MukB dimerization hinge (Nolivos et al., 2016), but as *matS* sites are spaced every ∼40 kbp and MatP does not spread from *matS* sites (Mercier et al., 2008), each MatP-*matS* complex must exert its effect distant from its site of binding on the chromosome, since the whole of *ter* is depleted for MukBEF complexes. We favor a process in which actively translocating MukBEF complexes dissociate from DNA when they encounter MatP-*matS* complexes, consistent with the observation that an ATP-hydrolysis-impaired MukB mutant remains associated with *matS* sites and *ter* (Nolivos et al., 2016). This also shows that the interaction of MukB with MatP-*matS* can occur in the absence of translocation but does not necessarily lead to dissociation from DNA.

Why does impairment of ATP-hydrolysis-dependent MukBEF action lead to defective segregation and the production of anucleate cells? In our opinion, lack of recruitment and catalytic activity stimulation of TopoIV by the MukB hinge interaction (Hayama and Marians, 2010, Li et al., 2010) is responsible for at least some of this phenotype. For example, delayed separation of newly replicated *oriC*s in Muk^−^ cells is thought to be a consequence of slower decatenation because of the absence of TopoIV from MukBEF clusters (Wang et al., 2008). Consistent with this, *mukBEF* deletion lowers the chromosome-bound fraction of the TopoIV subunit, ParC (Zawadzki et al., 2015). Since TopoIV, and likely MukBEF, are “multigate” protein complexes, it is possible that coordination between their actions occurs *in vivo*, consistent with the observed stimulation of TopoIV catalytic activity in the presence of MukBEF. The observed MukBEF axial cores are likely to be enriched for TopoIV, whose decatenase activity will then be directed to the bases of the loops emanating from the core. Cooperation in the action of SMC complexes and type II topoisomerases may also occur in other systems (Coelho et al., 2003, Uhlmann, 2016).

We conclude that chromosome-associated MukBEF complexes are the template and “machine” for formation of DNA loops in chromosomes, and their characterization here adds weight to the hypothesis that lengthwise compaction by intra-chromosome loop formation is the mechanism by which all SMC complexes organize and individualize chromosomes.

## STAR★Methods

### Key Resources Table

**Table:** 

REAGENT or RESOURCE	SOURCE	IDENTIFIER
**Bacterial Strains**		

*Escherichia coli* K12 AB1157	Bachmann, 1996	CGSC#1157
AB1157 (Ab246) *mukB(D1406A)-mYpet-kan*	Badrinarayanan et al., 2012a	N/A
AB1157 (SN191) *lacO*240-*hyg* at *L3* (2268 kb) *tetO*240*-gen* at *R3* (852 kb) Δ*leuB::P*_*lac*_*-lacI-mCherry-frt* Δ*galK::P*_*lac*_*-tetR-mCerulean-frt mukB-mYpet-frt*	Lab collection	N/A
AB1157 (SN192) *lacO*240-*hyg* at *ori1* (3908 kb) *tetO*240*-gen* at *ter3* (1644 kb) Δ*leuB::P*_*lac*_*-lacI-mCherry-frt* Δ*galK::P*_*lac*_*-tetR-mCerulean-frt mukB-mYpet-frt*	Nolivos et al., 2016	N/A
AB1157 (SN301) *lacO*240-*hyg* at *L3* (2268 kb) *tetO*240*-gen* at *R3* (852 kb) Δ*leuB::P*_*lac*_*-lacI-mCherry-frt* Δ*galK::P*_*lac*_*-tetR-mCerulean-frt mukB-mYpet-frt* Δ*matP::cat*	Lab collection	N/A
AB1157 (SN302) *lacO*240-*hyg* at *ori1* (3908 kb) *tetO*240*-gen* at *ter3* (1644 kb) Δ*leuB::P*_*lac*_*-lacI-mCherry-frt* Δ*galK::P*_*lac*_*-tetR-mCerulean-frt mukB-mYpet-frt* Δ*matP::cat*	Nolivos et al., 2016	N/A
AB1157 (SN311) *lacO*240-*hyg* at *ori1* (3908 kb) *tetO*240*-gen* at *ter3* (1644 kb) Δ*leuB::P*_*lac*_*-lacI-mCherry-frt* Δ*galK::P*_*lac*_*-tetR-mCerulean-frt mukB(E1407Q)-mYpet-kan*	Nolivos et al., 2016	N/A
AB1157 (JM41) *mukB-HaloTag-kan*	Banaz et al., 2019	N/A
AB1157 (JM56) *mukB-HaloTag-frt* Δ*mukE::kan*	This study	N/A
AB1157 (JM90) *lacO*240-*hyg* at *ori1* (3908 kb) *tetO*240*-gen* at *ter3* (1644 kb) Δ*leuB::P*_*lac*_*-lacI-mCherry-frt* Δ*galK::P*_*lac*_*-tetR-mCerulean-frt kan-araC-P*_*ara*_*-smtA-mukFE-mukB-mYpet-frt*	This study	N/A
AB1157 (JM91) *lacO*240-*hyg* at *ori1* (3908 kb) *tetO*240*-gen* at *ter3* (1644 kb) Δ*leuB::P*_*lac*_*-lacI-mCherry-frt* Δ*galK::P*_*lac*_*-tetR-mCerulean-frt kan-araC-P*_*ara*_*-smtA-mukFE-mukB-mYpet-frt,* Δ*matP::cat*	This study	N/A
AB1157 (JM97) *lacO*240-*hyg* at *ori1* (3908 kb) *tetO*240*-gen* at *ter3* (1644 kb) Δ*leuB::P*_*lac*_*-lacI-mCherry-frt* Δ*galK::P*_*lac*_*-tetR-mCerulean-frt frt-araC-P*_*ara*_*-smtA-mukFE-mukB(E1407Q)-mYpet-kan*	This study	N/A
AB1157 (JM101) *lacO*240-*hyg* at *L3* (2268 kb) *tetO*240*-gen* at *R3* (852 kb) Δ*leuB::P*_*lac*_*-lacI-mCherry-frt* Δ*galK::P*_*lac*_*-tetR-mCerulean-frt kan-araC-P*_*ara*_*-smtA-mukFE-mukB-mYpet-frt*	This study	N/A
AB1157 (JM103) *frt-araC-P*_*ara*_*-smtA-mukFE-mukB-HaloTag-kan*	This study	N/A
AB1157 (JM124) *lacO*240-*hyg* at *ori1* (3908 kb) *tetO*240*-gen* at *ter3* (1644 kb) Δ*leuB::P*_*lac*_*-lacI-mCherry-frt* Δ*galK::P*_*lac*_*-tetR-mCerulean-frt frt-araC-P*_*ara*_*-smtA-mukFE-mukB(D1406A)-mYpet-kan*	This study	N/A

**Chemicals, Peptides, and Recombinant Proteins**		

DL-serine hydroxamate	Sigma-Aldrich	Cat#S4503

**Deposited Data**		

Raw and analyzed data	This study	https://doi.org/10.17632/d7p7hk3zzv.1

**Oligonucleotides**		

PCR primers	Eurogentec	See Table S1

**Software and Algorithms**		

MATLAB	MathWorks	https://uk.mathworks.com/products/matlab.html
SuperSegger	Stylianidou et al., 2016	https://github.com/wiggins-lab/SuperSegger/wiki
NIS-Elements AR	Nikon	https://www.microscope.healthcare.nikon.com
SoftWoRx	GE Healthcare	https://www.gelifesciences.com/en/gb/shop/cell-imaging-and-analysis/high-and-super-resolution-microscopes/instruments/deltavision-omx-sr-imaging-system-p-03020
Chromagnon	Matsuda et al., 2018	https://github.com/macronucleus/chromagnon
ImageJ	Schneider et al., 2012	https://imagej.nih.gov/ij/
SIMcheck	Ball et al., 2015	https://github.com/MicronOxford/SIMcheck
3Dscript	Schmid et al., 2019	https://imagej.net/3Dscript
SGNS2	Lloyd-Price et al., 2012	https://sites.google.com/view/andreribeirolab/home/software

### Lead Contact and Materials Availability

The strains generated in this study are available without restriction. Further information and requests for resources should be directed to the Lead Contact, David J. Sherratt (david.sherratt@bioch.ox.ac.uk).

### Experimental Model and Subject Details

#### Bacterial strains and growth conditions

Bacterial strains and primers are listed in Key Resources Table and Table S1, respectively. All strains were derivatives of *E. coli* K12 AB1157 (Bachmann, 1996). *kan*, *cat*, *gen*, and *hyg* refer to insertions conferring resistance to kanamycin (Km^r^), chloramphenicol (Cm^r^), gentamycin (Gm^r^) and hygromycin B (Hyg^r^), respectively. The insertions are flanked by Flp site-specific recombination sites (*frt*) that allow removing the resistance gene using Flp recombinase from plasmid pCP20 (Datsenko and Wanner, 2000). To replace the native *smtA-mukBEF* promoter with an inducible *araC-P*_*ara*_, a sequence containing the *kan* resistance gene and *araC-P*_*ara*_ from the pBAD24 (Guzman et al., 1995) was constructed. Subsequently, the native promoter of *smtA-mukBEF* operon in strain SN192 was replaced by *kan-araC-P*_*ara*_ using λ-red recombination (Datsenko and Wanner, 2000). Finally, the generated chromosomal gene locus was transferred by phage P1 transduction to SN192 yielding strain JM90. P1 transduction was also used to introduce *kan-araC-P*_*ara*_*-smtA-mukFE-mukB-mYpet* into SN302, and SN191 resulting in strains JM91, and JM101, respectively. JM103 was constructed by first removing the *kan* resistance gene from JM90 using Flp recombinase, introducing *mukB-HaloTag-kan* from JM41 by λ-red recombination and transferring the generated chromosomal gene loci into AB1157 by P1 transduction. The JM56 strain was constructed by removing the *kan* resistance gene from JM41 and replacing the endogenous *mukE* gene with a kanamycin cassette using λ-red recombination. The JM97 strain was constructed by introducing *mukB(E1407Q)* mutation from SN311 by λ-red recombination into JM90 (after Flp recombinase), and verified by sequencing. The JM124 strain was constructed by introducing *mukB(D1406A)* mutation from Ab246 by λ-red recombination into JM90 (after Flp recombinase), and verified by sequencing. All genetic modifications were verified by PCR, the Muk^+/−^ phenotype was verified by temperature-resistance or lack of it in rich media, and behavior in quantitative imaging, as described (Nolivos et al., 2016).

Cells were grown in M9 minimal medium supplemented with 0.2% (v/v) glycerol, 2 μg ml^-1^ thiamine, and required amino acids (threonine, leucine, proline, histidine and arginine - 0.1 mg ml^-1^) at 30°C. For MukBEF overexpression strains, cells were additionally grown with a constant presence of 0.2% (w/v) L-(+)-arabinose. For microscopy, cells were grown overnight, diluted 1000-fold and grown to an A_600_ of 0.05–0.2. For anucleate cell percentages and DAPI intensity profile analysis, cells were stained with 1 μg/mL DAPI. Cells were then pelleted, spotted onto an M9 glycerol 1% (w/v) agarose pad on a slide and covered by a coverslip. For PALM microscopy, 0.17 mm thickness coverslips were plasma-cleaned of any background fluorescent particles before use. MukB-HaloTag was labeled with JF549 ligand (Grimm et al., 2015) as in (Banaz et al., 2019).

For experiments in which cells were enriched for completed non-replicating chromosomes, cells were treated with DL-serine hydroxamate (SHX) (final concentration of 1 mg ml^-1^). During the treatment, cells do not initiate new rounds of replication, but most complete any ongoing rounds (Ferullo et al., 2009). To allow sufficient time for ongoing replications to complete, cultures were grown in the presence of SHX for 3 h prior to imaging. This facilitated analysis of MukBEF axial cores and their distance relationships to genetic markers, because ongoing replication can bias results toward smaller *ori1* distances, as the number of *ori1* is greater than number of *ter3* (MatP^+^, *ori1*/*ter3* ratio 1.6; Δ*matP*, *ori1*/*ter3* ratio 1.5).

### Method Details

#### Epifluorescence microscopy

Fluorescence images were acquired on an inverted fluorescence microscope (Ti-E, Nikon) equipped with a perfect focus system, a 100× NA 1.4 oil immersion objective, a motorized stage, an sCMOS camera (Orca Flash 4, Hamamatsu), and a temperature chamber (Okolabs). Exposure times were 150 ms for mCherry and mYpet and 75 ms for mCerulean using an LED excitation source (Lumencor SpectraX).

Cell outlines, overall and per pixel MukB-mYPet fluorescence intensities, and FROS marker coordinates were detected using SuperSegger (Stylianidou et al., 2016) in MATLAB (MathWorks). The fraction of immature MukB-mYPet molecules was estimated by considering a maturation half time of 11.9 min for mYPet at 32°C (Balleza et al., 2018), cell generation time of 116 min, and that the MukB-mYpet expression level is in equilibrium. Consequently fluorescent molecules represent a fraction 1/(1+11.9 min/116 min) = 91% of the total MukB abundance. For fluorescence intensity profiles as a function of *ori1*/*ter3* distance (Figure 3E, F, Figure S3), cell pixel intensities were normalized by subtracting the average cell intensity and dividing by the maximum intensity. The distance from each pixel to the closest *ori1* and *ter3* markers were measured and the average intensity as a function of distance was calculated. For measurement of the angle between *ori1*/*ter3* markers and angular intensity profile for MukB (Figure S5), the center of the MukBEF structure was determined by separating MukB pixels belonging to the structure from the cellular background using Otsu’s thresholding (Otsu, 1979) and the center of mass of the region was estimated using *regionprops* (MATLAB). The angle between *ori1*/*ter3* markers was then calculated using the center of the structure. The angular intensity profile for MukB was measured by dividing the structure into 45 sectors using the center of the structure as the center point, calculating the background subtracted MukB structure pixel intensity in each sector, normalizing the intensities by the average sector intensity, and finally aligning the radial profile according to the *ori1*/*ter3* markers (Figure S5). For DAPI profiles (Figure S3), fluorescence intensity along the long cell axis for each cell was extracted. Only cells below 2.6 μm long were considered to avoid cells with more than one chromosomes. Both cell length and maximum fluorescence intensity were normalized, and the profiles were overlaid. DAPI area length was measured as full-width half-maximum of the DAPI profile.

#### Photoactivated localization microscopy

Live cell single-molecule-tracking photoactivated localization microscopy (PALM) was performed on a custom-built total internal reflection fluorescence (TIRF) microscope built around the Rapid Automated Modular Microscope (RAMM) System (ASI Imaging) with a motorized piezo stage, a z-motor objective mount, and autofocus system (MS-2000, PZ-2000FT, CRISP, ASI Imaging). MukB-HaloTag labeled with JF549 ligand was measured with a 100 mW 561 nm laser with 15% transmission (iChrome MLE, Toptica). The laser was collimated and focused through a 100× oil immersion objective (NA 1.4, Olympus) onto the sample using an angle for highly inclined thin illumination (Tokunaga et al., 2008). Fluorescence emission was filtered by a dichroic mirror and notch filter (ZT405/488/561rpc and ZET405/488/561NF, Chroma). Fluorescence emission was measured using an EMCCD camera (iXon Ultra, 512x512 pixels, Andor) with a pixel size of 96 nm. Transmission illumination was provided by an LED source and condenser (ASI Imaging). PALM movies were acquired with a frame time of 15.48 ms (Banaz et al., 2019).

Single molecule tracking data was analyzed using a custom-written MATLAB software (MathWorks) as in (Banaz et al., 2019, Stracy et al., 2019). Cell outlines were detected from bright-field images as in the previous section. Fluorescently-labeled MukB were detected by using band-pass filtering and an intensity threshold to each frame of the movie. These initial localizations positions were used as a start point in a two-dimensional elliptical Gaussian fit for a high-precision localization. Fitting parameters were x-position, y-position, x-width, y-width, elliptical rotation angle, intensity, and background. Single molecule tracking was performed by linking positions to a track if they appeared in consecutive frames within a window of 0.48 μm as in (Stracy et al., 2019). In rare cases of multiple localizations within the tracking radius, tracks were linked such that the sum of step distances was minimized. Tracking allowed for a transient (1 frame) disappearance of the molecule within a track due to blinking or missed localization. The mobility of each molecule was determined by calculating an apparent diffusion coefficient, D^∗^, from the stepwise mean-squared displacement (MSD) of the track using (Stracy et al., 2019):

$${\text{D}}^{*}=\frac{1}{4n\Delta t}\sum_{i=1}^{n}{\left[x\left(i\Delta t\right)-x\left(i\Delta t+\Delta t\right)\right]}^{2}+{\left[y\left(i\Delta t\right)-y\left(i\Delta t+\Delta t\right)\right]}^{2}$$

where *x(t)* and *y(t)* are the coordinates of the molecule at time *t*, the frame time of the camera is Δ*t*, and *n* is the number of the steps in the trajectory. Tracks shorter than  = 4 steps long were omitted due to the higher uncertainty in D^∗^. Threshold between mobile and immobile tracks was selected by measuring D^∗^ in Δ*mukE* strain (JM56) that does not stably associate with the chromosome and setting threshold to the lower 0.05 quantile of the D^∗^ distribution. Below this, threshold molecules were considered to be associated with the chromosome.

#### Measuring long-lasting binding events

PALM movies to measure long duration binding events of MukB-HaloTag labeled with JF549 dye were recorded using 1 s exposure times and low continuous 561 nm excitation (0.1% transmission) that blurs mobile molecules into the background whereas immobile molecules still appear as a diffraction-limited spot. Single molecule localization and tracking was used as described in the previous section. Additionally, bound and mobile molecules were distinguished by the width of the elliptical fits, with a short axis-width < 160 nm and long axis-width < 200 nm to determine bound molecules (Stracy et al., 2019) and missing frames were not allowed. The lengths of immobile tracks were measured and a survival probability curve (1-CDF) is shown in Figure 2D. To extract exponential-time constants, the survival probability curve of the immobile molecules was fitted to a double-exponential function corresponding to specific and non-specific DNA binding (Hansen et al., 2017, Rhodes et al., 2017) as a single-exponential function was found to not properly fit the survival probability curve. The fitting was performed using least-squares criterion with a weight 1/y to compensate for small values in the tail. The duration of specific DNA binding events was defined by the slower rate of the double-exponential function.

The probability of measuring a particular time of binding event is also influenced by the bleaching and blinking properties of the fluorescent dye. To assess the influence of these processes, along with errors in detection, the bleaching-time distributions were measured independently under the same conditions using cells fixed with 4% (v/v) paraformaldehyde that blocks molecule movement. As before, a bleaching-time survival probability curve was fitted by a double-exponential function to extract exponential-time constants. The MukB-HaloTag bleaching time constant, *t*_*bleach*_, was measured to be 48.8 ± 8.3 s (9739 tracks; ± SEM from 3 experiments). The bleaching corrected binding-time was calculated by *t*_*bound*_ = *t*_*measured*_^∗^ *t*_*bleach*_ / (*t*_*bleach*_ – *t*_*measured*_) (Rhodes et al., 2017). Blinking of fluorescent dye before or during binding events does not influence the measurement, because the observed binding times follow an exponential distribution and are therefore memoryless. All data analysis was performed in MATLAB (MathWorks).

#### 3D-structured illumination microscopy

Super-resolution 3D-structured illumination microscopy (SIM) images were acquired on a DeltaVision OMX V3 Blaze instrument (GE Healthcare), equipped with a 60× /1.42 oil UPlanSApo objective (Olympus), 405 nm, 488 nm and 593 nm diode lasers and three sCMOS cameras (PCO). Multiple-channel three-dimensional stacks of MukB–mYpet/TetR-mCerulean were imaged sequentially. For each channel, the raw 3D-SIM stacks were composed of 225 512x512 pixel images consisting of 21 z sections (125 nm z-spacing, sample thickness of 2.5 mm). Each section consisted of 15 images - 3 angles and 5 phase shifts. Additionally, LacI-mCherry was imaged in a conventional wide-field mode. Acquisition settings were as follows: MukB–mYpet, 20 ms exposure with 488 nm laser (attenuated to 30% transmission); TetR-mCerulean, 50 ms exposure with 405 nm laser (30% transmission), LacI-mCherry, 50 ms exposure with 593 nm laser (30% transmission). The 3D-SIM raw data was computationally reconstructed with SoftWoRx 6.0 (GE Healthcare) using a Wiener filter setting of 0.004 and channel specific optical transfer functions to generate a super-resolution three-dimensional image stack with a lateral (x–y) resolution of ∼120nm (wavelength dependent) and an axial (z) resolution of ∼300 nm. In the reconstruction process, the pixel size was halved from 80 nm to 40 nm and the pixel number doubled in order to meet the Nyquist sampling criterion. For multichannel 3D alignment, mouse C127 cells were three-color (405, 488 and 594 nm) 5-ethenyl-2′-deoxyuridine (EdU) pulse-labeled as described in (Kraus et al., 2017). The multichannel 3D-SIM EdU foci images were captured and reconstructed as described above, then channels were 3D aligned and corrected for chromatic shifts using the open-source software Chromagnon (Matsuda et al., 2018). The correction parameters obtained were then applied to align images from the experiments. ImageJ (Schneider et al., 2012) plugin 3Dscript (Schmid et al., 2019) was used to generate 3D rendering of MukB-mYpet signals (Videos S1 and S2).

For analysis of MukBEF structure dimensions (Figure 4D-F, Figure S4), 3D-SIM image stacks were processed using ImageJ plugin SIMcheck (Ball et al., 2015) and projected along the z axis and the maximum intensity for each pixel selected. Only filaments with clear orientation in xy-axes were selected for analysis. Pixels belonging to the MukBEF structure were separated from the background using Otsu’s thresholding (Otsu, 1979). The structure’s centerline was calculated by using a morphological operation (*bwmorph*, MATLAB, MathWorks) that erodes pixels from edges until only center pixels of the structure are left. Following this, branches of length 1 in the centerline were removed. The length of a linear structure was measured as the minimum length of a curve that includes all pixels of the centerline. The length of a circular structure was measured as the minimum contour length of a polygon that includes all pixels of the centerline. The thickness of the structure was measured by fitting a linegraph of pixel intensities across the centerline with a Gaussian function. Pixels close to the ends or branching points of the backbone were removed from the analysis. Further, the linegraph orientation was selected around a pixel to be normal to the structure so as to minimize width. From a Gaussian fit, full-width half-maximum (FWHM) distance was calculated as follows:

$$\text{FWHM}=2\sqrt{2\;\ln\;2\;}\sigma \approx 2.355\sigma$$

where σ is the standard deviation of the fitted Gaussian. All data analysis was performed in MATLAB (MathWorks).

#### Simulations of loop extrusion

Stochastic simulations were performed using SGNS2 (Lloyd-Price et al., 2012), which uses the Gillespie method (Stochastic Simulation Algorithm) (Gillespie, 1977) to obtain exact realizations of the Chemical Master Equation (CME). SGNS2 supports dynamic compartments that can be created or destroyed during a simulation. The circular chromosome of *E. coli* was divided into 4641 discrete DNA segments, with each segment corresponding to a specific 1 kbp region of the chromosome. MukBEF is, unless otherwise stated, modeled as a dimer of dimers, which randomly binds to 2 adjacent free sites on the chromosome with a stochastic rate (k_bind_) with equal probability throughout the chromosome. Binding of MukBEF creates a dynamic compartment that contains a single DNA loop where each dimer of MukBEF occupies a single DNA segment. Following the binding event, each dimer of the compartment moves unidirectionally and independently away from each other one DNA segment at a time (releasing previous DNA segment while occupying the consecutive one) with a stochastic rate (k_move_) for extrusion of a loop. As the MukBEF dimers move away from initial binding segment, DNA in the loop is free to be bound by other MukBEF molecules allowing for the creation of loops inside loops. If dimers collide on the chromosome head-on, they block each other, while the outer dimers of the MukBEF continue loop extrusion unperturbed. Unbinding of MukBEF releases the DNA segments under its footprint and destroys the loop with a stochastic rate (k_unbind_) that is independent of the state of the chromosome or other MukBEF. The residency time is the same everywhere on the chromosome, except in simulations with MatP dependent displacement of MukBEF from *ter* region, where binding or moving leads to instant dissociation of MukBEF molecule and destruction of the loop. Aforementioned reactions of the model are written for every DNA segment of the system with *ter* segments containing also the release reaction by MatP. Asymmetric loop extrusion is modeled by only one of the dimers moving away from the binding site; orientation decided randomly at the binding. The cytosolic state of MukBEF is assumed well-mixed and is therefore treated implicitly.

The rate constants were used as measured here. Namely, the MukBEF unbinding rate (k_unbind_) is 0.0154 s^-1^ (65 s residency time) and the MukBEF binding rate (k_bind_) is 3.9e-06 s^-1^ per DNA segment per free MukBEF complex which results in 48% of MukBEF to be bound to the chromosome with wild-type MukBEF copy numbers (110 MukBEF dimer of dimers) (Badrinarayanan et al., 2012a). The loop extrusion rate (k_move_) has not been directly measured *in vivo* and therefore was set to 0.6 DNA segments/dimer/s (corresponding to 600 bp/dimer/s) as estimated *in vitro* for condensin (Ganji et al., 2018). The expected loop size without collisions is 80 kbp (40 kbp for unidirectional loop extrusion). Simulations for loop extrusion rates of 100 bp/dimer/s and 1500 bp/dimer/s were also undertaken. The system state including the state of each loop compartment was read out after 500 s to allow the overall loop structure on the chromosome to reach maturation. Each simulation was repeated at least 1000 times to ensure proper sampling of chromosome states.

In the analysis of simulated chromosomes, MukBEF clusters were defined as MukBEF complexes that do not have unlooped DNA segments between them. Loops inside loops can contribute to the cluster size if they have reached the stem of the main loop by at least from one side. After finding the largest MukBEF cluster, the shortest distance between the largest cluster and a chromosome locus was measured along the chromosome from a DNA segment of the specific chromosome locus to the closest MukBEF of the largest cluster. Loops acts as ‘shortcuts’ decreasing the distances between chromosomal loci. The loop state of a single chromosome (Figure 5C) was shown as a polar coordinate plot that shows the starting and the ending locations of DNA loops or as a 2D force-directed layout of the circular chromosome after converting the loop state into a graph with loops as connections between otherwise circular organization of DNA segments. Random links (Figure 5A) were generated by adding 52 (wild-type occupancy) connections between random DNA segments. All data analysis was performed in MATLAB (MathWorks).

### Quantification and Statistical Analysis

Statistical details of experiments can be found in the figure legends. This includes exact value of samples, number of experiments and definition of dispersion measures (SD or SEM) between experiments. Microscopy images were randomly collected to obtain sufficient number of cells for each dataset. No data was excluded besides the specific criteria defined in the figure legends. Independent experiments were used to define the reproducibility of results. Two-sample unpaired Student’s t test, performed by using *ttest2* function in MATLAB (MathWorks), was used for hypothesis testing of equal means and equal but unknown variances between samples. Significance was defined as p value < 0.01.

### Data and Code Availability

The following data are available at Mendeley data (https://doi.org/10.17632/d7p7hk3zzv.1): The raw epifluorescence data (Figures 3, 6, S1, S3, and S5); the single-molecule localizations and tracks used for D^∗^ histograms and residency times (Figure 2); and the raw data for representative images in all figures. All materials and codes are available upon reasonable request.

## Supplemental Information

Document S1. Figures S1–S6 and Table S1

Document S2. Article plus Supplemental Information
