# Supplementary material for: Organization of the Escherichia coli Chromosome by a MukBEF Axial Core
Source: Mol Cell. 2020 Apr 16;78(2):250–260.e5. doi: 10.1016/j.molcel.2020.02.003 (PMC7163298; doi:10.1016/j.molcel.2020.02.003)
Supplement: Document S1. Figures S1–S6 and Table S1 [file mmc1.pdf]

Molecular Cell, Volume *78*

Supplemental Information

Organization of the *Escherichia coli* Chromosome by a MukBEF Axial Core

Jarno Mäkelä and David J. Sherratt

Figure S1

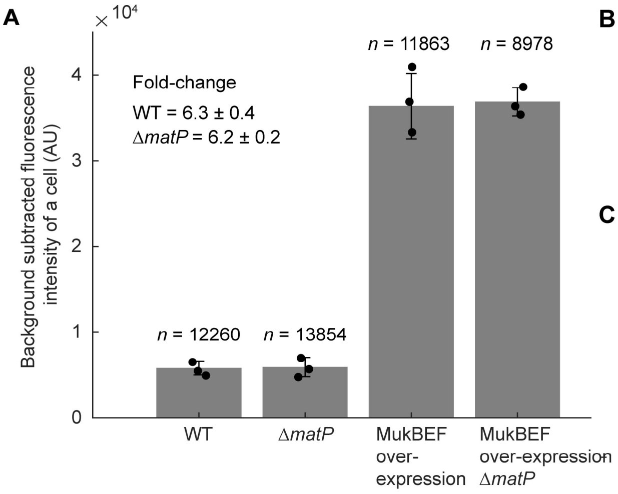

B

Generation times:

Wild type	116 ± 4 min
MukBEF over-expression	115 ± 3 min
MukBEF over-expression *ΔmatP*	116 ± 1 min

C

Anucleate cells:

Wild type (8726 cells)	0.23 ± 0.01%
ΔmatP (9223 cells)	0.66 ± 0.27%
MukBEF over-expression (24537 cells)	0.12 ± 0.06%
MukBEF over-expression *ΔmatP* (19409 cells)	0.59 ± 0.16%

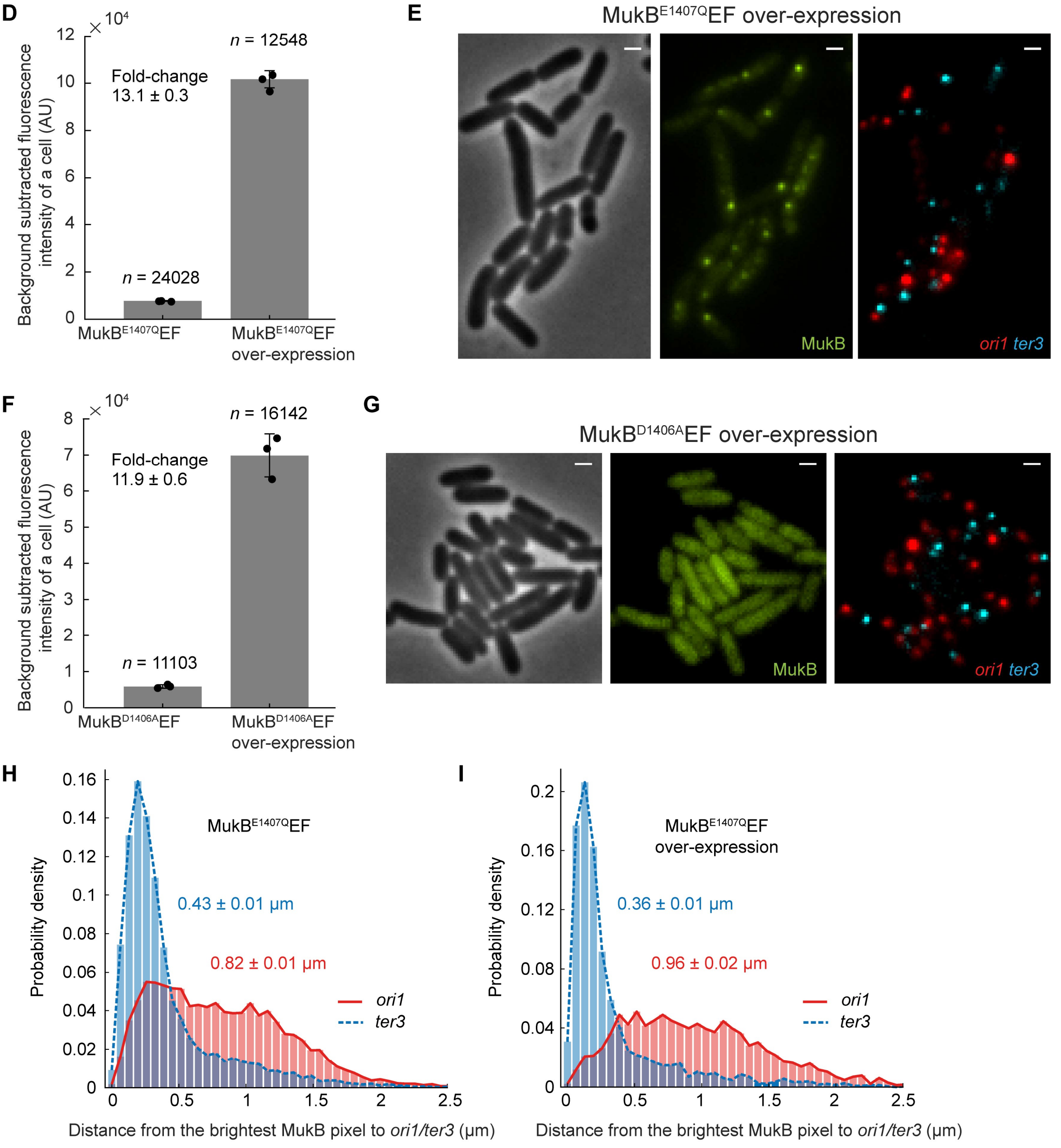

Figure S1. Characterization of increased MukBEF chromosome occupancy cells and MukBEF mutants. Related to Figure 1.

(**A**) MukB-mYpet fluorescence intensity from 3 repeats in WT, Δ*matP*, MukBEF over-expression and MukBEF over-expression Δ*matP* cells and fold-change between WT and MukBEF over-expression. *n* denotes number of cells and error bars denote SD. (**B**) Generation times from 3 repeats (±SEM) for WT, over-expression and over-expression Δ*matP*. (**C**) Anucleate cell percentages using DAPI from 3 repeats (±SEM) in WT, Δ*matP*, MukBEF over-expression and MukBEF over-expression Δ*matP*. (**D**) MukB^{E1407Q}-mYpet fluorescence intensity in MukB^{E1407Q}EF and MukB^{E1407Q}EF over-expression cells. *n* denotes number of cells and error bars denote SD from 3 repeats. (**E**) Representative phase contrast and fluorescence images of MukB^{E1407Q}EF over-expression cells with *ori1* and *ter3* markers. Scale bars, 1 µm. (**F**) MukB^{D1406A}-mYpet fluorescence intensity in MukB^{D1406A}EF and MukB^{D1406A}EF over-expression cells. *n* denotes number of cells and error bars denote SD from 3 repeats. (**G**) Representative phase contrast and fluorescence images of MukB^{D1406A}EF over-expression cells with *ori1* and *ter3* markers. Scale bars, 1 µm. (**H**)-(**I**) Distances between the brightest MukB^{E1407Q}-mYpet pixel and *ori1*/*ter3* markers in (**H**) MukB^{E1407Q}EF (8773 cells) and (**I**) MukB^{E1407Q}EF over-expression cells (2313 cells). Data from 3 repeats (±SEM).

Figure S2

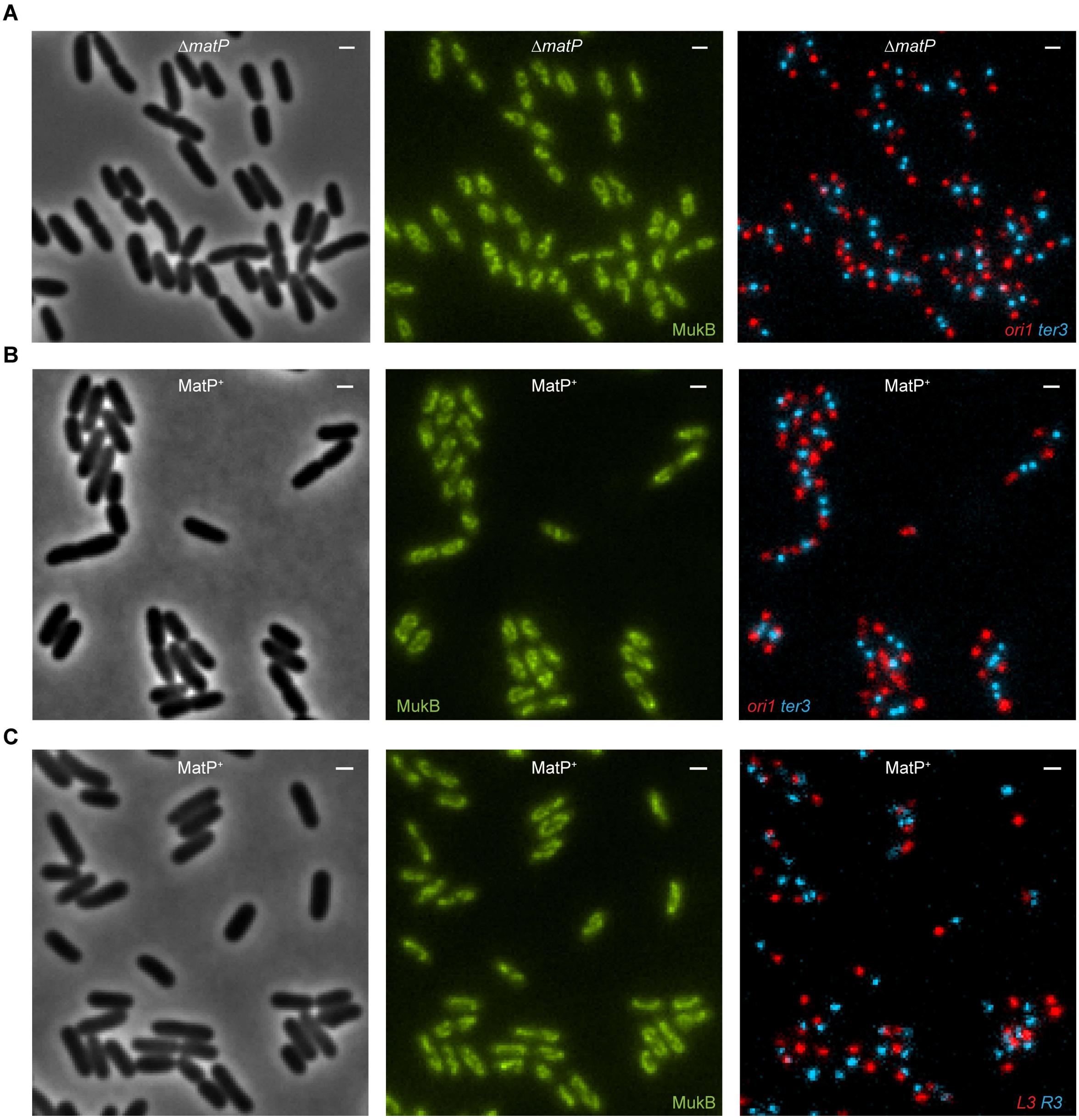
A
ΔmatP
ΔmatP
ΔmatP
MukB
ori1 ter3
B
MatP⁺
MatP⁺
MatP⁺
MukB
ori1 ter3
C
MatP⁺
MatP⁺
MatP⁺
MukB
L3 R3

Figure S2. MukBEF increased occupancy cells in MatP^{+} (with MatP present) and Δ*matP*. Related to Figure 1.

Representative phase contrast and fluorescence images of cells with (**A**) MukBEF increased occupancy Δ*matP* cells with *ori1* and *ter3* markers, (**B**) MukBEF increased occupancy with *ori1* and *ter3* markers, and (**C**) MukBEF increased occupancy with *L3* and *R3* markers. Scale bars, 1 μm.

Figure S3

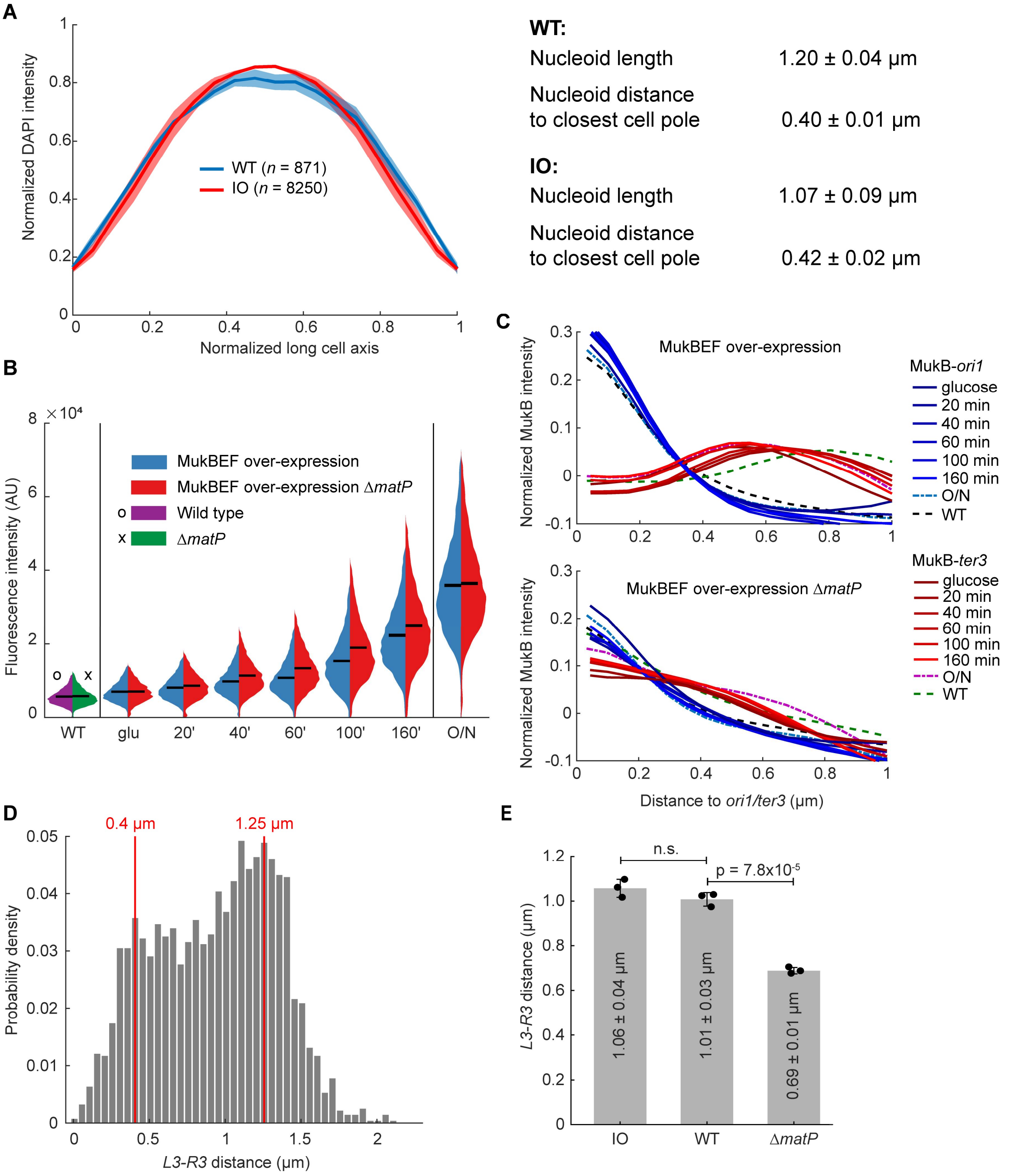

A
Normalized DAPI intensity
Normalized long cell axis
WT (n = 871)
IO (n = 8250)
WT:
Nucleoid length 1.20 ± 0.04 µm
Nucleoid distance to closest cell pole 0.40 ± 0.01 µm
IO:
Nucleoid length 1.07 ± 0.09 µm
Nucleoid distance to closest cell pole 0.42 ± 0.02 µm
B
Fluorescence intensity (AU)
×10⁴
MukBEF over-expression
MukBEF over-expression ΔmatP
o Wild type
x ΔmatP
WT
glu
20'
40'
60'
100'
160'
O/N
C
MukBEF over-expression
MukBEF over-expression ΔmatP
Normalized MukB intensity
MukB-ori1
glucose
20 min
40 min
60 min
100 min
160 min
O/N
WT
MukB-ter3
Distance to ori1/ter3 (µm)
D
0.4 µm
1.25 µm
Probability density
L3-R3 distance (µm)
E
n.s.
p = 7.8x10-5
L3-R3 distance (µm)
1.06 ± 0.04 µm
1.01 ± 0.03 µm
0.69 ± 0.01 µm
IO
WT
ΔmatP

Figure S3. DAPI profiles, induction of MukBEF over-expression time series and *L3*-*R3* distances. Related to Figure 1, 2, and 4.

(**A**) Normalized DAPI intensity profiles on normalized long cell axis for WT (871 cells) and MukBEF increased occupancy (8250 cells). Only cells below 2.6 µm long were considered to avoid cells with more than 1 chromosomes. Nucleoid length was measured as full-width-half-maximum (FWHM) of the DAPI profile. Also, the distance to the cell pole from half maximum of the DAPI profile was measured. Shaded area denotes SEM. Data are from 3 repeats. (**B**) MukB-mYpet intensity in wild type and MukBEF over-expression cells during induction with 0.2 % (w/v) arabinose. Fluorescence expression levels are shown prior to induction (glucose), during induction (20 min, 40 min, 60 min, 100 min, 160 min) and cultures grown over night in the presence of arabinose. Left distributions correspond to MukBEF increased occupancy MatP$^+$ strain and right distributions to the MukBEF increased occupancy Δ*matP* strain. Also shown are wild type and Δ*matP* strains. Black lines show the means. Data are from at least 2 independent experiments with number of data points in WT (12260, 13854), glucose (9537, 4614), 20 min (8521, 9385), 40 min (11419, 5939), 60 min (8982, 8995), 100 min (9630, 10152), 160 min (9396, 10212), and over-night induction (11863, 8979) in MatP$^+$ and Δ*matP* cells, respectively (no. cells). (**C**) Normalized MukB-mYpet pixel intensity during induction as a function of distance to *ori1*/*ter3* in (top) MukBEF increased occupancy and (bottom) MukBEF increased occupancy Δ*matP* cells. Also shown are wild type and Δ*matP* strain. Data are same as in (B). (**D**) Distances between *L3* and *R3* markers in MukBEF increased occupancy cells. 2824 single chromosome cells from 2 experiments were analyzed. (**E**) Distances between *L3* and *R3* markers in IO (7467 cells), WT (2444 cells) and Δ*matP* (2796 cells) cells. Prior to imaging cells were treated with serine hydroxamate and only cells with a single chromosome were analyzed. Two-sample t-test was used to compare conditions. Error bars denote SD from 3 experiments.

Figure S4

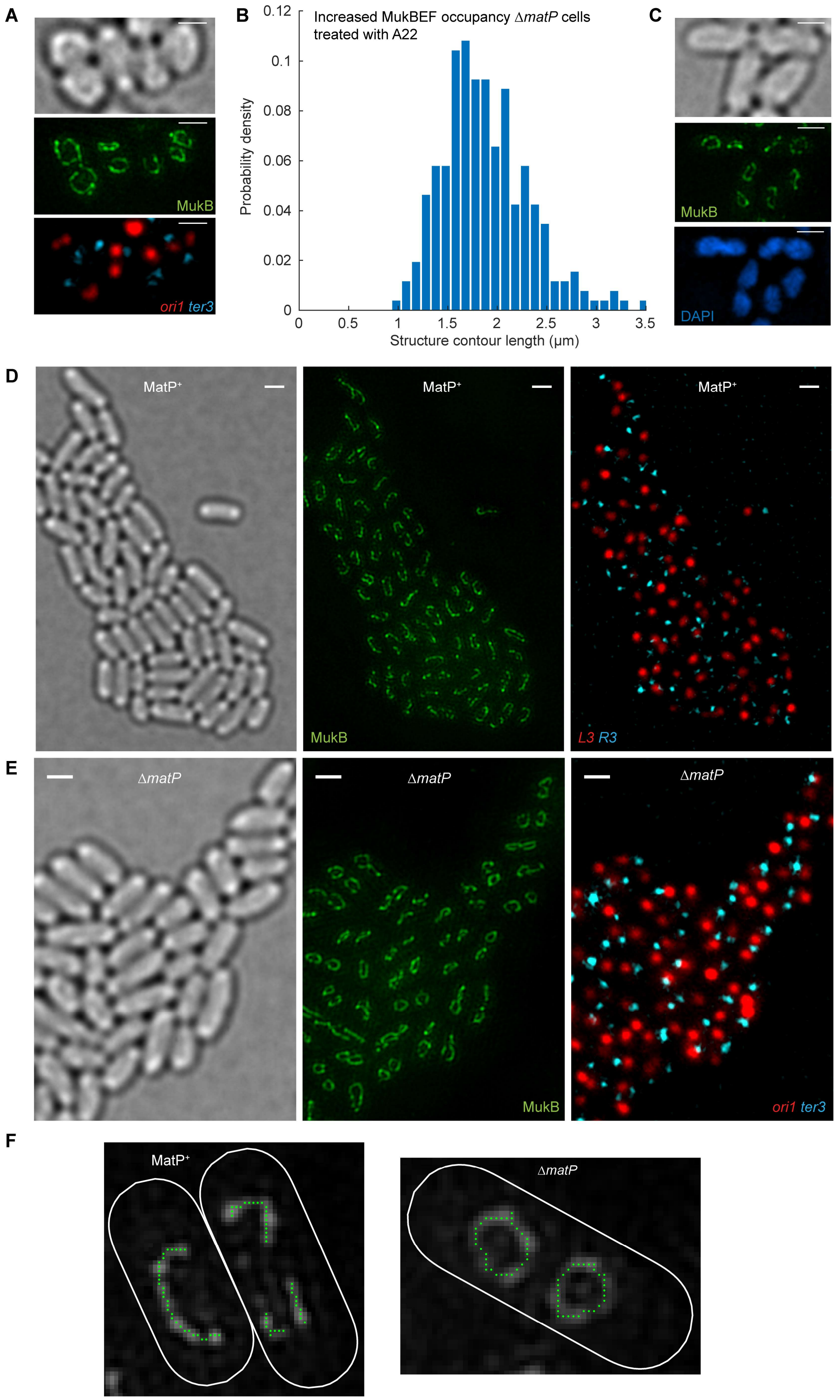

A
MukB
ori1 ter3
B
Increased MukBEF occupancy ΔmatP cells treated with A22
Probability density
Structure contour length (μm)
0.12
0.1
0.08
0.06
0.04
0.02
0
0
0.5
1
1.5
2
2.5
3
3.5
C
MukB
DAPI
D
MatP+
MukB
L3 R3
E
ΔmatP
MukB
ori1 ter3
F
MatP+
ΔmatP

Figure S4. 3D-SIM images of IO cells and IO cells treated with A22 or rifampicin. Related to Figure 1, 4 and STAR Methods.

(**A**) Representative SIM images of MukBEF increased occupancy Δ*matP* cells with *ori1* and *ter3* markers grown with A22 (4 μg/ml) for 6 h. 3D-SIM images were maximum projected onto 2D for visualization purposes. Scale bars, 1 μm. (**B**) Distribution of axial core contour lengths (259 chromosomes) in the same conditions as in (A). (**C**) Representative SIM images of MukBEF increased occupancy Δ*matP* cells with *ori1* and *ter3* markers grown with rifampicin (0.025 μg/ml) for 2 h. Scale bars are 1 μm. Representative SIM images of MukBEF increased occupancy in (**D**) $MatP^+$ cells with *L3* and *R3* markers, and (**E**) Δ*matP* cells with *ori1* and *ter3* markers. 3D-SIM images were maximum projected onto 2D for visualization. Scale bars, 1 μm. (**F**) Example images of detected centerlines of circular and linear the MukBEF structures in Δ*matP* and $MatP^+$ cells, respectively. White line is the cell border. Green dots are centerline pixels of the structure.

Figure S5

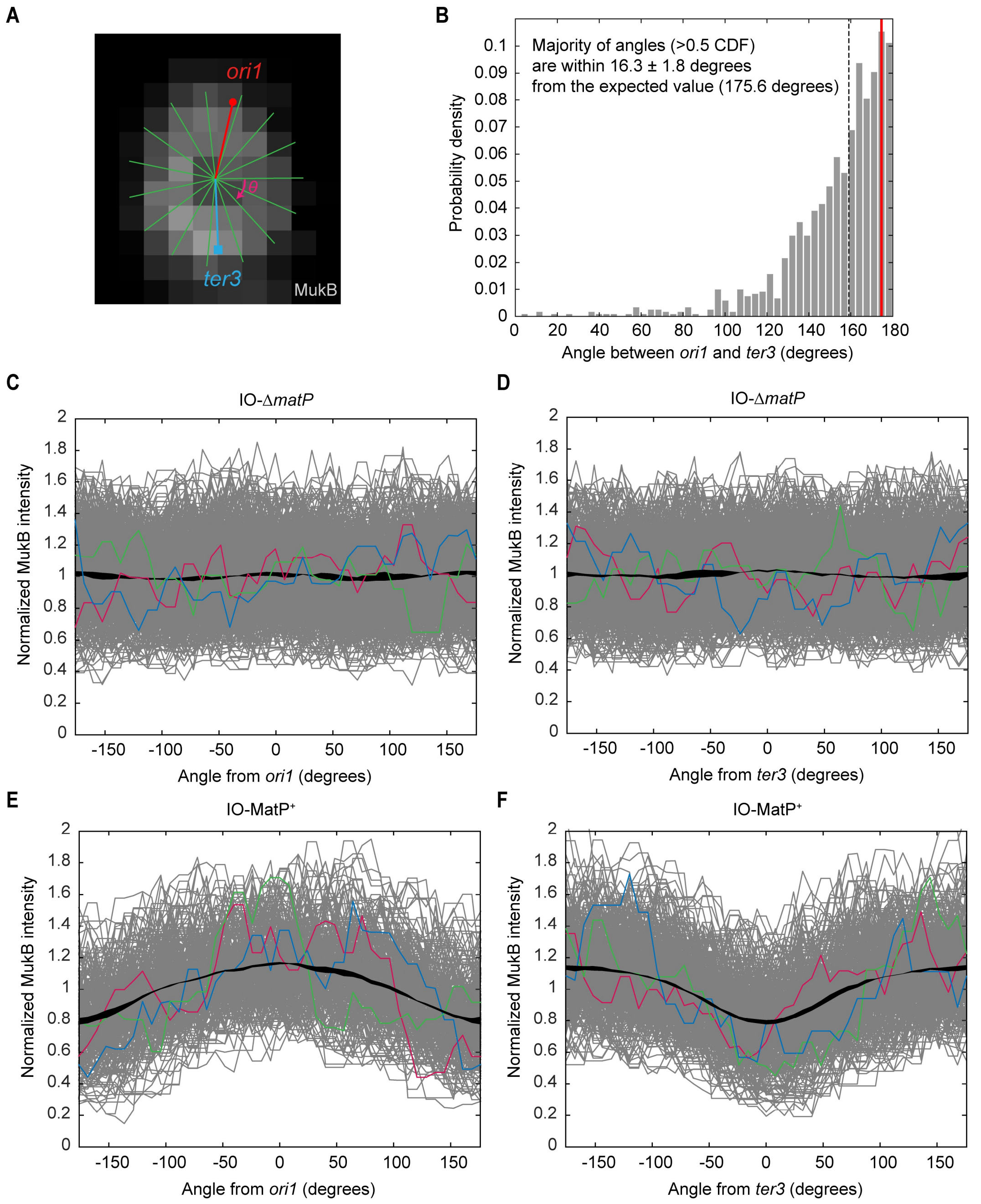
A
ori1
θ
ter3
MukB
B
Majority of angles (>0.5 CDF)
are within 16.3 ± 1.8 degrees
from the expected value (175.6 degrees)
Probability density
Angle between ori1 and ter3 (degrees)
C
IO-ΔmatP
Normalized MukB intensity
Angle from ori1 (degrees)
D
IO-ΔmatP
Normalized MukB intensity
Angle from ter3 (degrees)
E
IO-MatP+
Normalized MukB intensity
Angle from ori1 (degrees)
F
IO-MatP+
Normalized MukB intensity
Angle from ter3 (degrees)

Figure S5. MukBEF axial core analysis. Related to Figure 3.

(**A**) Illustration of a circular axial core divided into sectors with *ori1* and *ter3* markers. Using the center of the axial core, the angle between *ori1* and *ter3* is measured. The axial core is divided into sectors from which the MukB intensity is calculated (in the actual measurement 45 sectors were used). (**B**) The minimum angle between *ori1* and *ter3* in IO-Δ*matP* cells (1206 cells from 3 repeats). Only cells with a single chromosome (a single *ori1* and *ter3*) and symmetric structures are included in the analysis (relative difference between long and short axis less than 0.3). Prior to imaging, cells were treated with SHX. The red line is the expected angle (175.6 degrees) between *ori1* and *ter3*. (**C**)-(**F**) Normalized radial MukBEF intensity of axial cores in (**C**) IO-Δ*matP* cells aligned to *ori1*, (**D**) IO-Δ*matP* cells aligned to *ter3*, (**E**) IO-MatP$^+$ cells aligned to *ori1*, and (**F**) IO-MatP$^+$ cells aligned to *ter3*. Black line shows the mean with the thickness corresponding to ±SD. Colored lines are representative axial core intensities. The intensity is measured only for the pixels contained in the MukBEF structure. Prior to imaging, cells were treated with serine hydroxamate. Only cells with a single chromosome (a single *ori1* and *ter3*) and a symmetric structure are included in the analysis (difference between long and short axis less than 0.3). Data are from 3 repeats (IO-Δ*matP* 1206 cells; IO-MatP$^+$ 1495 cells).

Figure S6

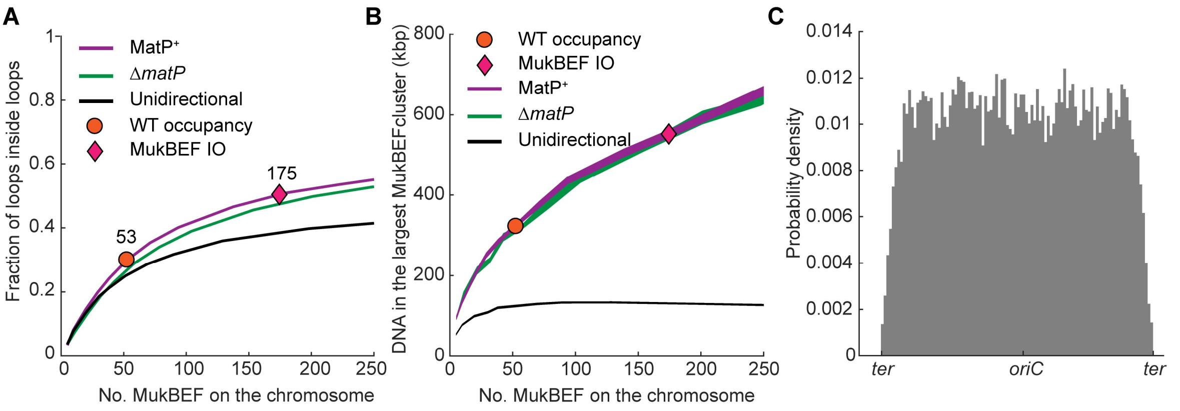

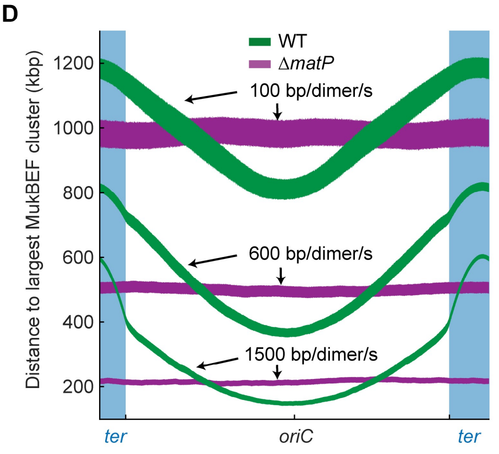

E

100 bp/dimer/s	Loop size (kbp)	Fraction of DNA in loops
WT	13.69 (13.63-13.74)	0.150 (0.149-0.150)
Δ*matP*	13.78 (13.72-13.84)	0.161 (0.164-0.166)
OE	11.33 (11.30-11.37)	0.384 (0.383-0.386)
OE-Δ*matP*	11.62 (11.59-11.66)	0.439 (0.438-0.441)
**600 bp/dimer/s**		
WT	52.16 (51.93-52.38)	0.48 (0.477-0.481)
Δ*matP*	54.44 (54.21-54.68)	0.548 (0.546-0.551)
OE	30.44 (30.35-30.53)	0.726 (0.725-0.727)
OE-Δ*matP*	31.87 (31.78-31.95)	0.858 (0.857-0.860)
**1500 bp/dimer/s**		
WT	87.71 (87.34-88.06)	0.661 (0.659-0.663)
Δ*matP*	93.99 (93.62-94.32)	0.773 (0.770-0.775)
OE	42.51 (42.40-42.61)	0.792 (0.791-0.793)
OE-Δ*matP*	45.54 (45.43-45.67)	0.946 (0.944-0.947)

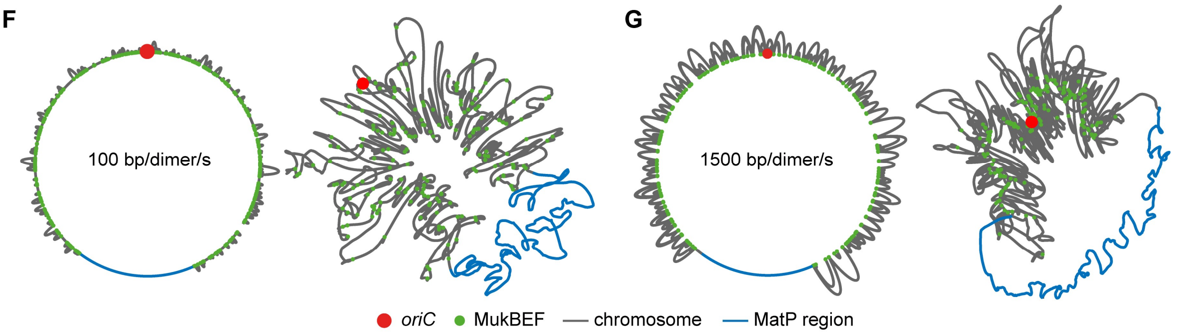

Figure S6. Modeling loop extrusion by MukBEF analysis. Related to Figure 5.

(**A**) Fraction of loops inside loops and (**B**) DNA in the largest MukBEF cluster (no unlooped DNA between them) as function of the number of bound MukBEF dimers of dimers. Experimentally observed numbers of MukBEF dimers of dimers on the chromosome for wild-type (○) and MukBEF increased occupancy (◊) are depicted. Additionally, unidirectional model of loop extrusion (black line) is shown where each dimers binds and extrudes a loop independently in a randomly chosen direction. Line thickness denotes 95% bootstrap confidence interval for the mean across at least 1000 simulation replicas. (**C**) MukB occupancy profile on the chromosome with wild-type MukBEF occupancy across 4000 simulation replicas. (**D**) Shortest distance from a chromosome locus to the largest MukBEF cluster with different loop extrusion rates (100 bp/dimer/s, 600 bp/dimer/s and 1500 bp/dimer/s) with (WT) or without (Δ*matP*) MukBEF displacement from *ter*. Line thickness denotes 95% bootstrap confidence interval for the mean across 2000 simulation replicas. (**E**) Average loop size and fraction of the chromosome in loops for different loop extrusion rates. Expected loop sizes without collisions are 15 kbp, 80 kbp and 197 kbp for 100 bp/dimer/s, 600 bp/dimer/s, and 1500 bp/dimer/s, respectively. 95% bootstrap confidence interval for the mean across 1000 simulation replicas in parenthesis. (**F**)-(**G**) Representative *E. coli* chromosomes for (**F**) 100 bp/dimer/s and (**G**) 1500 bp/dimer/s loop extrusion rates with increased MukBEF occupancy. (left) Beginning and end of loops with MukBEF (green dots) along the chromosome. (right) Force-directed layouts of the chromosomes.

Table S1. Primers used for strain construction. Related to STAR Methods.

Name	Sequence	Construct
OL1_F	GGATTCTGCAAACCCTATGCTACTCCCGGAGTGTAGGCTGGAGCTGCTTC	*kan-araC-*P_{ara} construction using Gibson assembly. PCR on pBAD24 (OL3_F/OL2_R) and pKD4 OL1_F/OL4_R).
OL2_R	GAAGCAGCTCCAGCCTACACTCCGGGAGTAGCATAGGGTTTGCAGAATCC	
OL3_F	TAAGGAGGATATTCATATGGGTAACCGTCAAGCCGTCAATTGTCTGATTC	
OL4_R	GAATCAGACAATTGACGGCTTGACGGTTACCCATATGAATATCCTCCTTA	
OL5_F	CCACAGCAGCGCCAGGCCAGCGCCAATAATCAACAACATCAGCGGAAGTGAGTGTAGGCTGGAGCTGCTTC	λ-red replacement of P_{muk} with *kan-araC-*P_{ara} at the endogenous locus. PCR on *kan-araC-*P_{ara} from Gibson.
OL6_R	TGTAATATCGCTGGCGATCCCTTGCTATATGGTTAAAAAAGGAACCAGAAGAATTCCTCCTGCTAGCCCAAAA	
OL7_F	AATTGTGTGAGCGTTTGCAAATGCA	λ-red insertion of *mukB-HaloTag-kan*. PCR on JM41.
OL8_R	GTACAACGCCAATACTCACGAAAGT	
OL9_F	CCGATTAATGATTACGGAGCCA	λ-red deletion of *mukE*. PCR on strain with Δ*mukE::kan* from lab collection.
OL10_R	CCCGGCTTCCGTAGTGTTACGGAAA	
OL11_F	GTATCGTTTGGTCAGGTGAACAG	λ-red insertion of *mukB(E1407Q)-mYpet-kan* or *mukB(D1406A)-mYpet-kan*. PCR on SN311 or Ab246.
OL12_R	GTACAACGCCAATACTCACGAAAGT	
